# Detailed morphology of tentacular apparatus and central nervous system in *Owenia borealis* (Annelida, Oweniidae)

**DOI:** 10.1186/s40851-021-00182-y

**Published:** 2021-12-05

**Authors:** Elena Temereva, Nadezhda Rimskaya-Korsakova, Vyacheslav Dyachuk

**Affiliations:** 1 Department of Invertebrate Zoology, Biological Faculty, Moscow State University, Lomonosov State University, Leninskie Gory 1, bld. 12, Moscow, 119992 Russia; 2 National Scientific Center of Marine Biology, Far Eastern Branch, Russian Academy of Sciences, Vladivostok, 690041 Russia

**Keywords:** medullary dorsal commissure, stratified neuroepithelium, tentacle, coelom, dorsal root of circumesophageal connective, antenna

## Abstract

The Oweniidae are marine annelids with many unusual features of organ system, development, morphology, and ultrastructure. Together with magelonids, oweniids have been placed within the Palaeoannelida, a sister group to all remaining annelids. The study of this group may increase our understanding of the early evolution of annelids (including their radiation and diversification). In the current research, the morphology and ulta-anatomy of the head region of *Owenia borealis* is studied by scanning electron microscopy (SEM), 3D reconstructions, transmission electron microscopy (TEM), and whole-mount immunostaining with confocal laser scanning microscopy. According to SEM, the tentacle apparatus consists of 8–14 branched arms, which are covered by monociliary cells that form a ciliary groove extending along the oral side of the arm base. Each tentacle contains a coelomic cavity with a network of blood capillaries. Monociliary myoepithelial cells of the tentacle coelomic cavity form both the longitudinal and the transverse muscles. The structure of this myoepithelium is intermediate between a simple and pseudo-stratified myoepithelium. Overall, tentacles lack prominent zonality, i.e., co-localization of ciliary zones, neurite bundles, and muscles. This organization, which indicates a non-specialized tentacle crown in *O. borealis* and other oweniids with tentacles, may be ancestral for annelids. TEM, light, and confocal laser scanning microscopy revealed that the head region contains the anterior nerve center comprising of outer and inner (=circumoral) nerve rings. Both nerve rings are organized as concentrated nerve plexus, which contains perikarya and neurites extending between basal projections of epithelial cells (radial glia). The outer nerve ring gives rise to several thick neurite bundles, which branch and extend along aboral side of each tentacle. Accordingly to their immunoreactivity, both rings of the anterior nerve center could be homologized with the dorsal roots of circumesophageal connectives of the typical annelids. Accordingly to its ultrastructure, the outer nerve ring of *O. borealis* and so-called brain of other oweniids can not be regarded as a typical brain, i.e. the most anterior ganglion, because it lacks ganglionic structure.

## Background

The Annelida is a speciose and morphologically diverse evolutionary lineage belonging to the larger animal clade Lophotrochozoa. Annelids exhibit extremely wide patterns of organ system anatomy and ultrastructure [1]. According to recent data, the Annelida can be divided into two large clades, Errantia and Sedentaria, and also includes several groups, so-called basal branching lineages, including oweniids, chaetopterids, amphinomids, sipunculids, etc. [2–7]. Members of the family Oweniidae have many unusual morphological, ultrastructural, and developmental characteristics [8–16]. Oweniids together with magelonids have been recently placed among the Palaeoannelida, a sister group to all remaining annelids [5, 7]. The study of oweniids may increase our understanding of the evolution of annelids, especially their early radiation and diversification. New data on oweniid morphology can help to reconstruct the annelid common ancestor, which together with comparative data across bilaterians may contribute to inferring character states in the last common bilaterian ancestor (LCBA).

In particular, new data are important for resolution of problem of ancestral organization of the nervous system in Bilateria. At present, there are two main hypotheses regarding the structure of the anterior nerve center of the LCBA: it consisted of either a ganglionic accumulation of neurons or a diffuse nerve plexus [17, 18]. The first hypothesis suggests that the LCBA could have simple ganglia, or even an elaborated brain, defined as a central collection of neuronal centers with distributed and hierarchical functions [19, 20]. The organizations of the ganglia and brains have been well studied [21–23]. The second hypothesis suggests that the anterior nerve center is organized as a nerve plexus, or a non-ganglionic intraepidermal anterior nerve center [7, 15, 23–30].

Another one interesting question concerning the LCBA is whether it had tentacle-like appendages. In extant metazoans, tentacles are used for food collection by cnidarians and ctenophores, as well as by many bilaterian groups including phoronids, brachiopods, bryozoans, entoprocts, annelids, mollusks, hemichordates, echinoderms, and chordates [1, 31]. The presence of tentacles in many groups suggests that the LCBA may have also had tentacles. If tentacles are inherited from the LCBA, they must have evolved in different directions among bilaterians. Although the directions of tentacle evolution remain uncertain, we know that some organisms have specialized tentacles [32–38]. This specialization is expressed in the zonation and co-localization of several organ systems: ciliary bands, nerve cords, and muscles [39–47]. Such specialized tentacles are present in the lophophorates [48–51]. To increase our understanding of how tentacles have evolved among the Bilateria, we require detailed data on the organization and development of tentacles from different groups of recent bilaterians.

In the current report, we provide a detailed description of the anatomy and ultra-anatomy of the head and tentacle apparatus of *Owenia borealis*. Since oweniids occupy basal position within annelid phylogeny, have an intraepidermal non-ganglionic nerve center composed of stratified neuroephitelium [11–13, 15, 26], and might have (Owenia and Myriowenia) or not have tentacles (Galathowenia and Myriochele) [52] they remain a crucial taxon in understanding evolution of nervous system and tentacles evolution in annelids. Considering that the morphology of oweniids is highly relevant to discussions of the last common ancestor of Annelida, we discuss our data in the broader context of annelid evolution.

## Materials and Methods

About 20 adults of *Owenia borealis* Koh, Bhaud & Jirkov, 2003 [53] were collected in September 2018 near the Espegrend Marine Biological Station, University of Bergen, Norway. Live adults were extracted from their tubes and were used for the research.

### Scanning electron microscopy (SEM)

The structure of the head was studied by scanning electron microscopy (SEM). The heads of three specimens were postfixed in 1% OsO4 and dehydrated in an ascending ethanol and acetone series, critical point dried, and then sputter coated with platinum-palladium. Specimens were examined with a JEOL JSM-6380LA (JEOL Ltd., Tokyo, Japan) microscope at operating voltages of 15–20 kV at Lomonosov Moscow State University.

### Transmission electron microscopy (TEM)

The head regions with tentacles were fixed overnight at 4 °C in a 2.5% solution of glutaraldehyde in 0.2 M phosphate buffer (PBS). The heads were then washed in 0.2 M PBS for 4 h with three changes and postfixed in 1% OsO_4_ in 0.2 M PBS for 3 h at room temperature (RT) with gentle rotation. The specimens were then dehydrated in an increasing series of ethanol concentrations (from 15 to 96%) and isopropanol. They were subsequently infiltrated in a mixture of isopropanol and Spurr resin for 3 days and then embedded in pure Spurr resin at 60 °C for 24 h.

The anterior part of the body of two adults embedded in resin were used to prepare a complete series of 1-μm (semi-thin) and 70-nm (thin) resin sections with a Leica UC 7 ultramicrotome (Leica Microsystems, Wetzlar, Germany). The semi-thin sections were stained with methylene blue and examined with a Zeiss Axioplan2 light microscope equipped with an AxioCam HRm camera (Carl Zeiss Microscopy, LLC, USA). Semi-thin sections were used for description of gross anatomy and for 3D reconstructions. The thin sections were stained with uranyl acetate and lead citrate and were examined with a JEM-1011 JEOL or a JEM-100 B-1 JEOL transmission electron microscope (JEOL, Akishima, Japan).

### Whole-mount immunostaining and confocal laser scanning microscopy (CLSM)

Adults were fixed in a 4% paraformaldehyde solution in PBS (pH 7.4) (ThermoFisher Scientific, Pittsburgh, PA, USA) for 8 h at 4 °C and then were washed three times (30 min each time) in PBS with 1% Triton X-100 (PBT) (ThermoFisher Scientific). The specimens were then placed in a mixture of normal goat serum and PBT (NGS 15%) for 2 h to block the sites of unspecific staining. For immunostaining, solution of primary antibodies (Abs) rabbit anti 5-HT (Immunostar, 20,080, 1:1000) alone or in combination with mouse anti acetylated-alpha-tubulin (Santa Cruz, sc-23,950, 1:1000) in PBT were used. The animals were incubated in primary Abs for 24 h at 4 °C with rotation, followed by triple rinses with PBT. The secondary antibody mixtures consisted of donkey anti-rabbit 488 (Life Technologies, A21206, 1:1000) with donkey anti-mouse 555 (Life Technologies, A31572, 1:1000) in PBT, for 24 h at 4 °C. After antibodies labeling, specimens were placed in 1:100 dilution of 4′, 6-diamidino-2-phenylindole (DAPI, Molecular Probes, USA,) in PBT for 4 h at RT. As a control for non-specific immunorecognition, we performed immunohistochemical staining without the primary antibodies, adding only the secondary antibodies or normal (non-immunized) immunoglobulin G (1:500–1:1000; Sigma-Aldrich; I5006, I5381). The specimens were then washed three times in PBS, washed for several minutes in increasing concentrations of isopropanol, and embedded in Murray Clear (a 50/50 mixture of benzyl benzoate and benzyl alcohol) at RT. Specimens were observed with a Zeiss LSM 780 confocal microscope (Far Eastern Center of Electron Microscopy, A.V. Zhirmunsky National Scientific Center of Marine Biology, Far Eastern Branch of the Russian Academy of Sciences, Vladivostok, Russia) and with with a Nikon Eclipse Ti confocal microscope (Nikon Corporation, Tokyo, Japan) at Lomonosov Moscow State University, Moscow, Russia.

### Image processing

Z-projections were prepared using ImageJ software [54]. Volume renderings were prepared with Amira version 5.2.2 software (ThermoFisher Scientific, MA, USA). Images were processed in Adobe Photoshop CS3 (Adobe Systems, San Jose, CA, USA). Three-demensional reconstructions were prepared with Imaris 7.2.1 software (ThermoFisher Scientific, MA, USA).

## Results

### Morphology of the head and tentacles

**Fig. 1:**
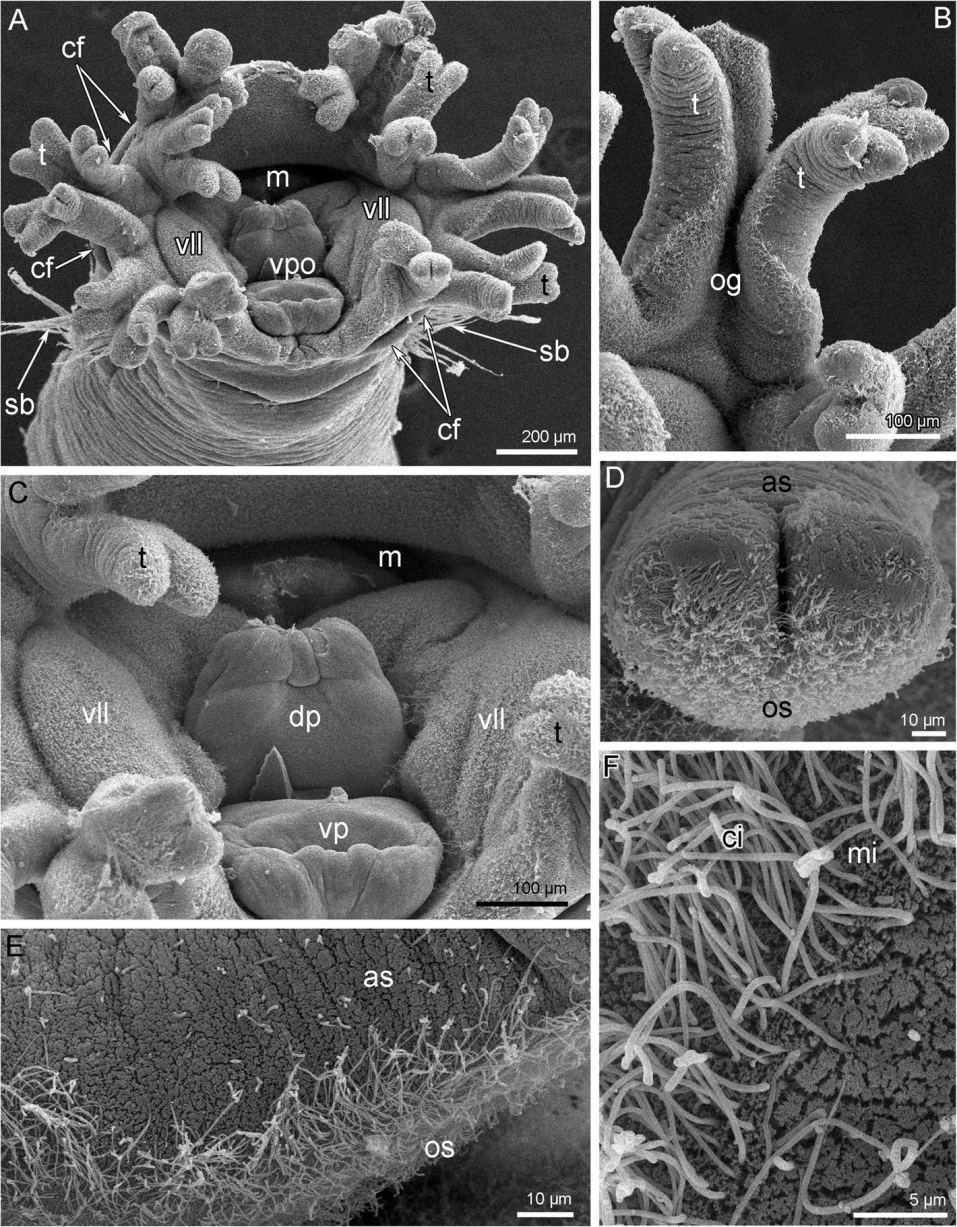
Morphology of the head and tentacles of *Owenia borealis* (SEM). **A** The head viewed from the top. **B** Tentacle. **C** Ventral pharyngeal organ. **D** Forked tip of tentacle. **E** Ciliated oral and non-ciliated aboral sides of tentacle. **F** A portion of the oral side of tentacle. Abbreviations: as – aboral side; cf. – collar fold; ci – cilia; dp – dorsal part of pharyngeal organ; m – mouth; mi – microvilli; og – oral groove; os – oral side; sb – setae bundle; t – tentacle; vll – ventrolateral lip; vp – ventral part of pharyngeal organ; vpo – ventral pharyngeal organ

**Fig. 2:**
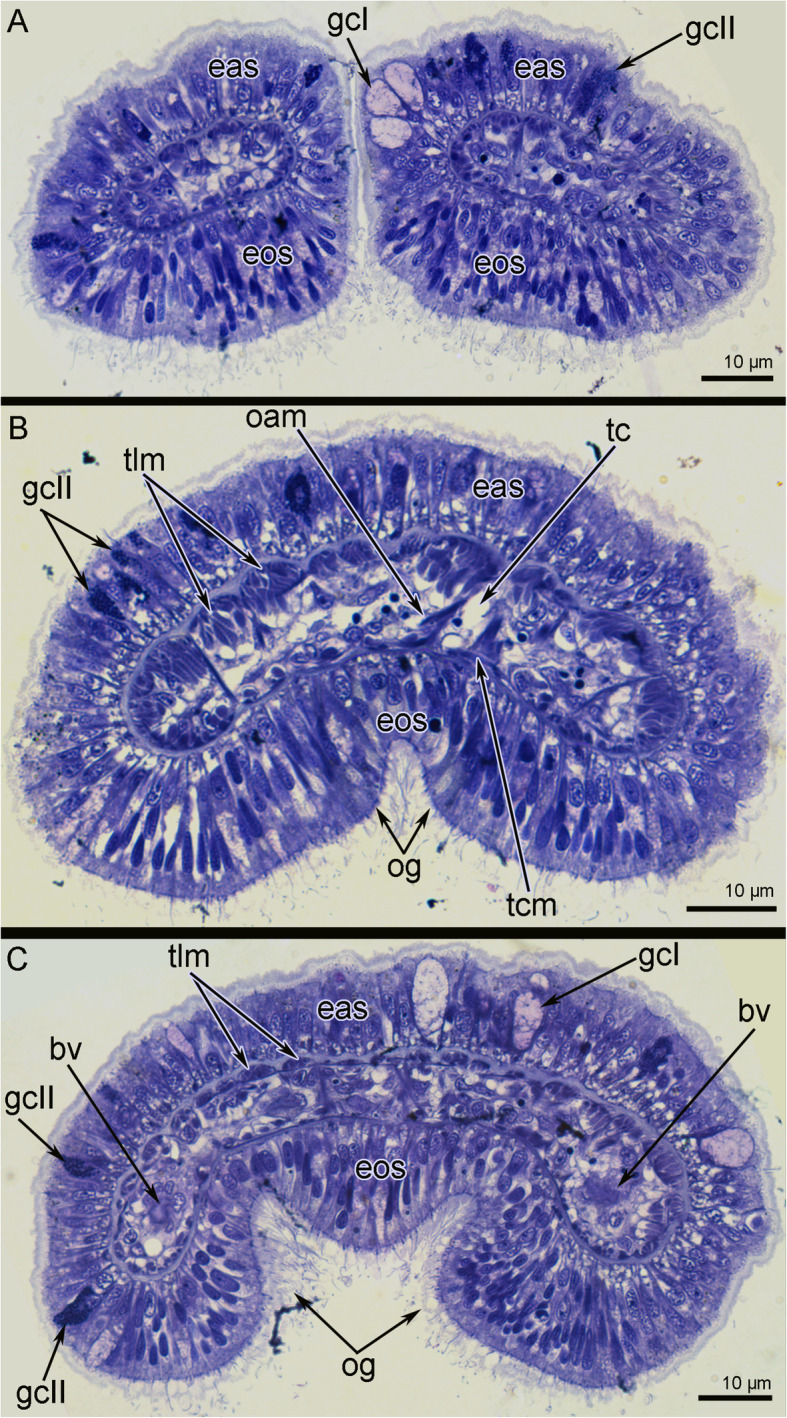
Organization of tentacles of *Owenia borealis*. Transverse semi-thin sections at different levels of tentacles. **A** Forked tip of tentacle. **B** Middle portion of tentacle. **C** Base of tentacle. Abbreviations: bv – blood vessel; eas – epithelium of aboral side; eos – epithelium of oral side; gcI – gland cell of first type; gcII – gland cell of second type; oam – oral-aboral muscles; og – oral groove; tc – tentacle coelom; tcm – tentacle transverse muscle; tlm – tentacle longitudinal muscles

The tentacle crown of *O. borealis* bears two lateral groups of arms, which are separated on the dorsal and ventral sides (Fig. 1A). The number of arms can vary in different specimens: therefore each lateral group consists of four, five or seven arms. Each arm branches on two tentacles, which then bifurcate and form two short bulbs with small Y-shaped tips on each (Figs. 1B, D; 2A). Tentacles and arms are covered by cilia, which are abundant on the oral side and are almost absent on the aboral side (Fig. 1D-F, 2A). The base of the tentacles has a deep groove extending along the oral side (Fig. 2B, C). This groove is prominent at the base of the tentacle arm (Fig. 2C). The base of the tentacle crown forms a collar that extends along the external side of the head (Fig. 1C). The ventral pharyngeal organ, consisting of the dorsal and ventral lips, is very large and is located at the ventral side of the tentacle crown. Two ventrolateral lips are adjacent to the ventral pharyngeal organ and are covered by cilia (Fig. 1C). The mouth resembles a crescent slit (Fig. 1A). The base of the tentacle apparatus is surrounded by a thin collar fold from the outside of the tentacle crown (Fig. 1A).

### Histology and ultrastructure of the tentacles and head

#### Epithelium

**Fig. 3:**
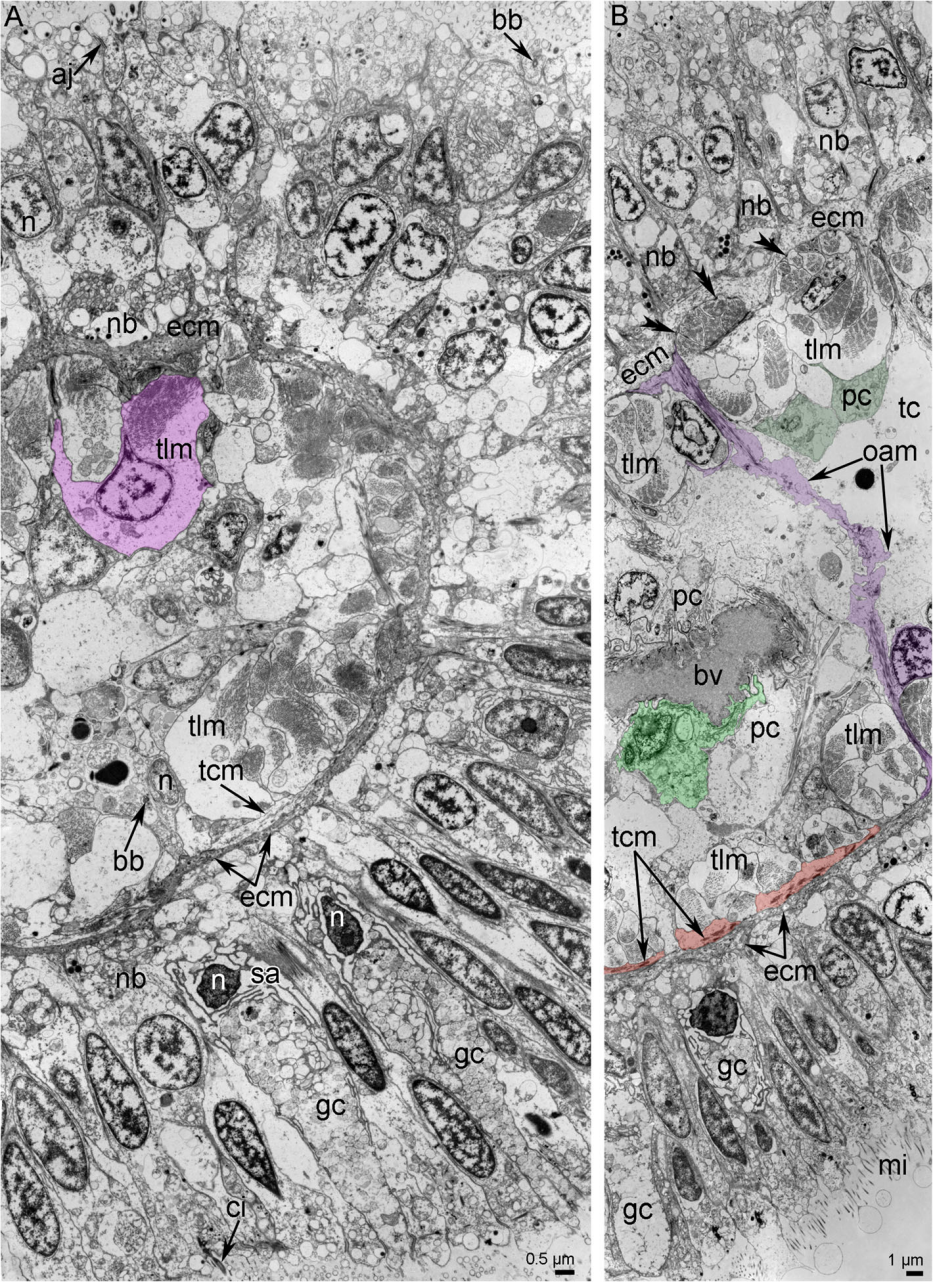
Details of tentacle ultrastructure of *Owenia borealis* (TEM). **A** General view of epithelia and coelomic cavity of a tentacle. A cell of longitudinal muscle is shown in pink. **B** Different tentacle muscles: oral-aboral muscle is shown in violet, tentacle cross muscles are shown in orange. Peritoneal cells, which cover longitudinal muscles, are shown in dark green. A cell of the wall of blood vessel is shown in light green. Hemidesmosomes are indicated by double arrowheads. Abbreviations: aj – adherence junction; bb – basal body; bv – blood vessel; ci – cilium; ecm – extracellular matrix; gc – gland cell; mi – microvilli; n – nucleus; nb – neurite bundle; oam – oral-aboral muscle; pc – peritoneal cells; sa – secretory area of the gland cell; tcm – tentacle transverse muscles; tlm – tentacle longitudinal muscles

**Fig. 4:**
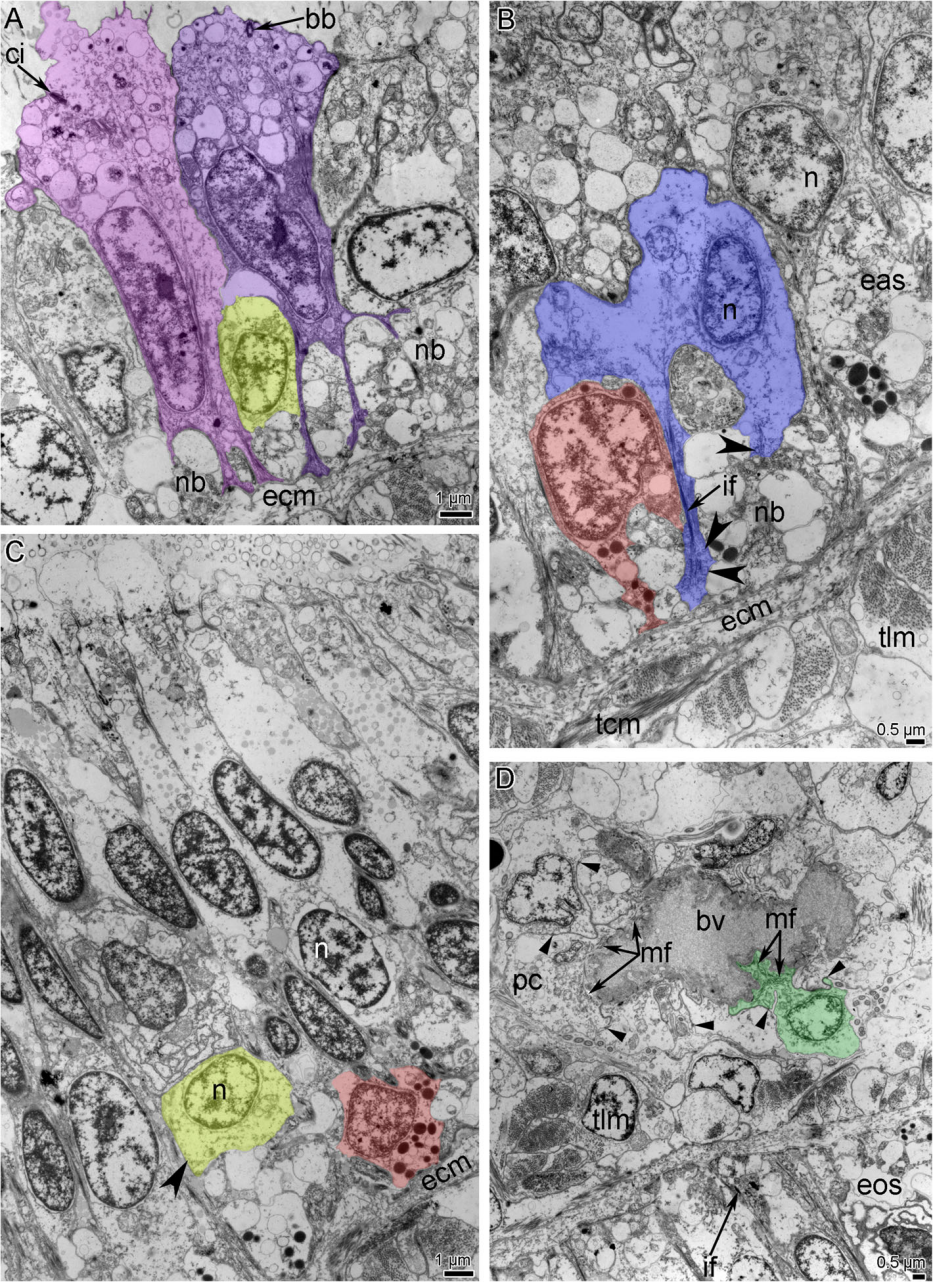
Ultrastructure of tentacle nerves and blood vessel of *Owenia borealis* (TEM). **A** Epithelium of aboral side: different types of cells. Two supportive cells are shown by different colors; perikaryon is shown in yellow. **B** The base of epithelium of aboral side: two types of glial cells are shown by red and blue. Vesicles in glial cell are indicated by concaved arrowheads. **C** Epithelium of oral side with perikaryon (yellow) and glial cell (red). Vesicles in perikaryon are indicated by concaved arrowheads. **D** Myoepithelial cells (green) of coelomic lining, which form the wall of blood vessel. Adherence junctions between cells are indicated by straight arrowheads. Abbreviations: bb – basal body; bv – blood vessel; ci – cilium; ecm – extracellular matrix; eas – epithelium of aboral side; eos – epithelium of oral side; if – intermediate filaments; mf – myofilaments; n – nucleus; nb – neurite bundle; pc – peritoneal cells; tcm – tentacle transverse muscles; tlm – tentacle longitudinal muscles

Each tentacle is covered by ciliated epithelial cells and contains a coelomic cavity that contains muscles and blood vessels (Figs. 2C, 3A, B). The aboral epithelium is formed by large monociliated cells, which are filled with many vesicles of different diameter (Fig. 4A). These cells form the basal thin projections that contain electron-dense filaments, surround the neurite bundles, and attach to the basal lamina (Fig. 4A). Large gland cells of different types are scattered in the epithelium of the aboral side (Fig. 2A, C). Some of these cells have large vacuoles and electron-lucent content (Fig. 2A, C), and others have many small and dense granules in the cytoplasm (Fig. 2). Longitudinal neurite bundles extend along the base of the aboral epithelium, which also contains cells with different organization (Fig. 4A, B). Between epithelial cells small roundish perikarya are scattered (Fig. 4A). Some basal cells contain ovoid electron-dense granules, therefore their projections with the same granules can be easily recognized between neurite bundles (Fig. 4B). Other cells form large thin and thick projections, which contain electron-dense intermediate filaments and synaptic vesicles (Fig. 4B).

The epithelium of the oral side consists of slender high cells, which bear a cilium and do not form prominent basal projections (Fig. 4C). The cytoplasm of these cells contains prominent, apical transverse and longitudinal electron-dense fibers. The epithelium of the oral side contains many glandular cells, whose cytoplasm is filled with roundish vesicles with flocculent content (Fig. 3A). The secretory apparatus and nucleus are located in the basal part of the glandular cells. Neurite bundles, which extend between the basal parts of the epithelial cells, are less numerous than in the aboral epithelium (Fig. 4C). Perikarya and cells with ovoid electron-dense granules are scattered in the basal portion of the epithelium of the oral side (Fig. 4C).

The epithelium lies on the extracellular matrix layer (ECM) (Fig. 3A, B). The aboral ECM is up to 2 μm thick (Fig. 3B). The ECM is 2–3 times thinner on the oral side than on the aboral side of the tentacle (Fig. 3A).

#### Coelomic cavity and musculature

**Fig. 5:**
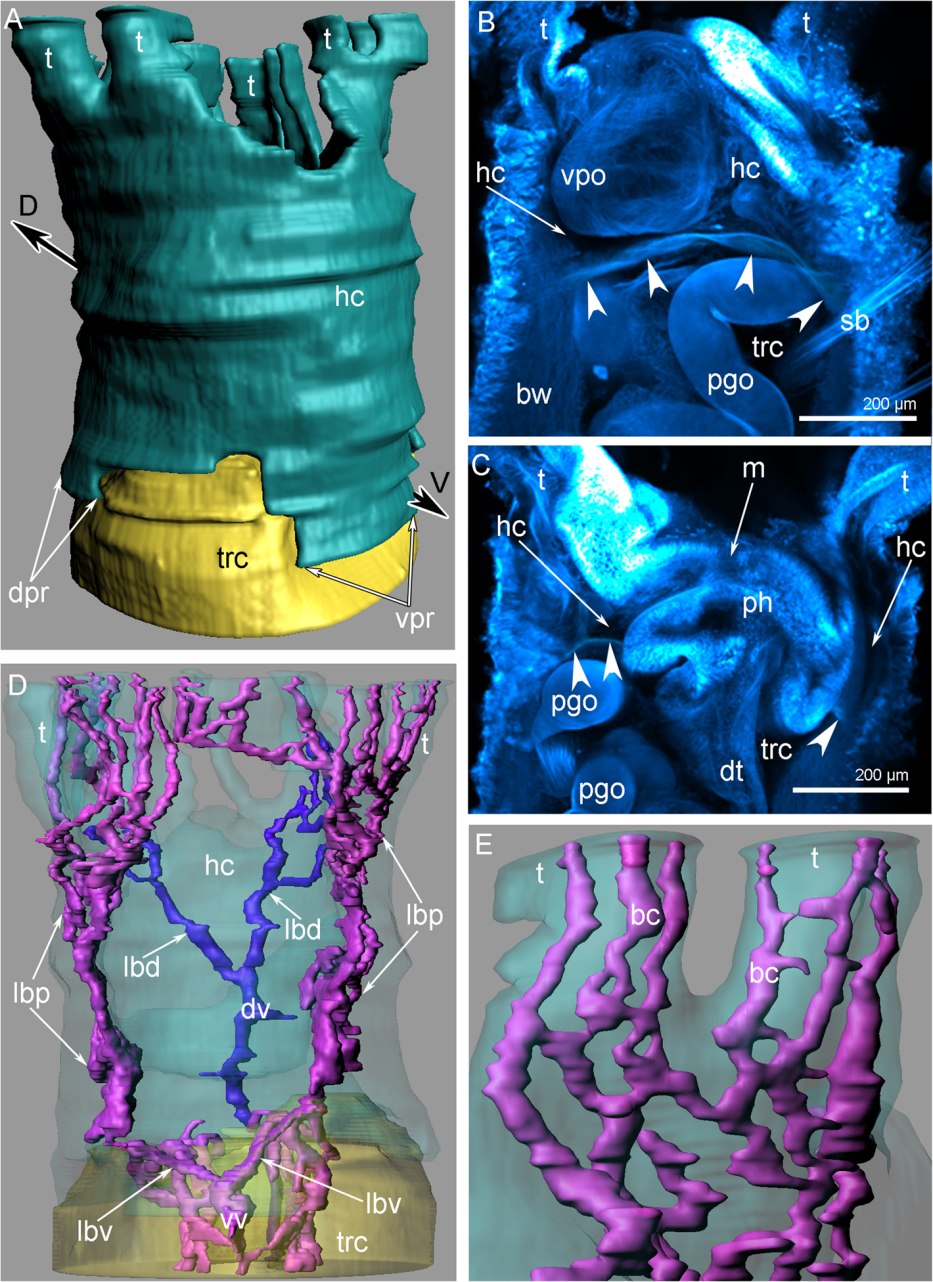
Organization of the coelom of the head of *Owenia borealis*. **A** Three-dimensional reconstruction of head (green) and trunk (yellow) coeloms. **B** Z-projection of head after staining with DAPI: dissepiment between head and trunk coeloms is indicated by arrowheads. **C** Z-projection of head and trunk cavity at sagittal optical section after staining with DAPI: dissepiment between head and trunk coeloms is indicated by arrowheads. **D** Three-dimensional reconstruction of blood vessels in head and part of trunk. Trunk and head coeloms are partly transparent. **E** Three-dimensional reconstruction of blood capillaries in tentacles. Abbreviations: bc – blood capillary; bw – body wall; dpr – dorsal protrusion; dt – digestive tube; dv – dorsal blood vessel; hc – head coelom; lbd – lateral branch of dorsal blood vessel; lbv – lateral branch of ventral blood vessel; lbp – lateral blood plexus; m – mouth; pgo – parapodial glandular organ; ph – parynx; t – tentacle; sb – setae bundle; trc – trunk coelom; vpo – ventral pharyngeal organ; vpr – ventral protrusion of the head coelom; vv – ventral blood vessel

**Fig. 6:**
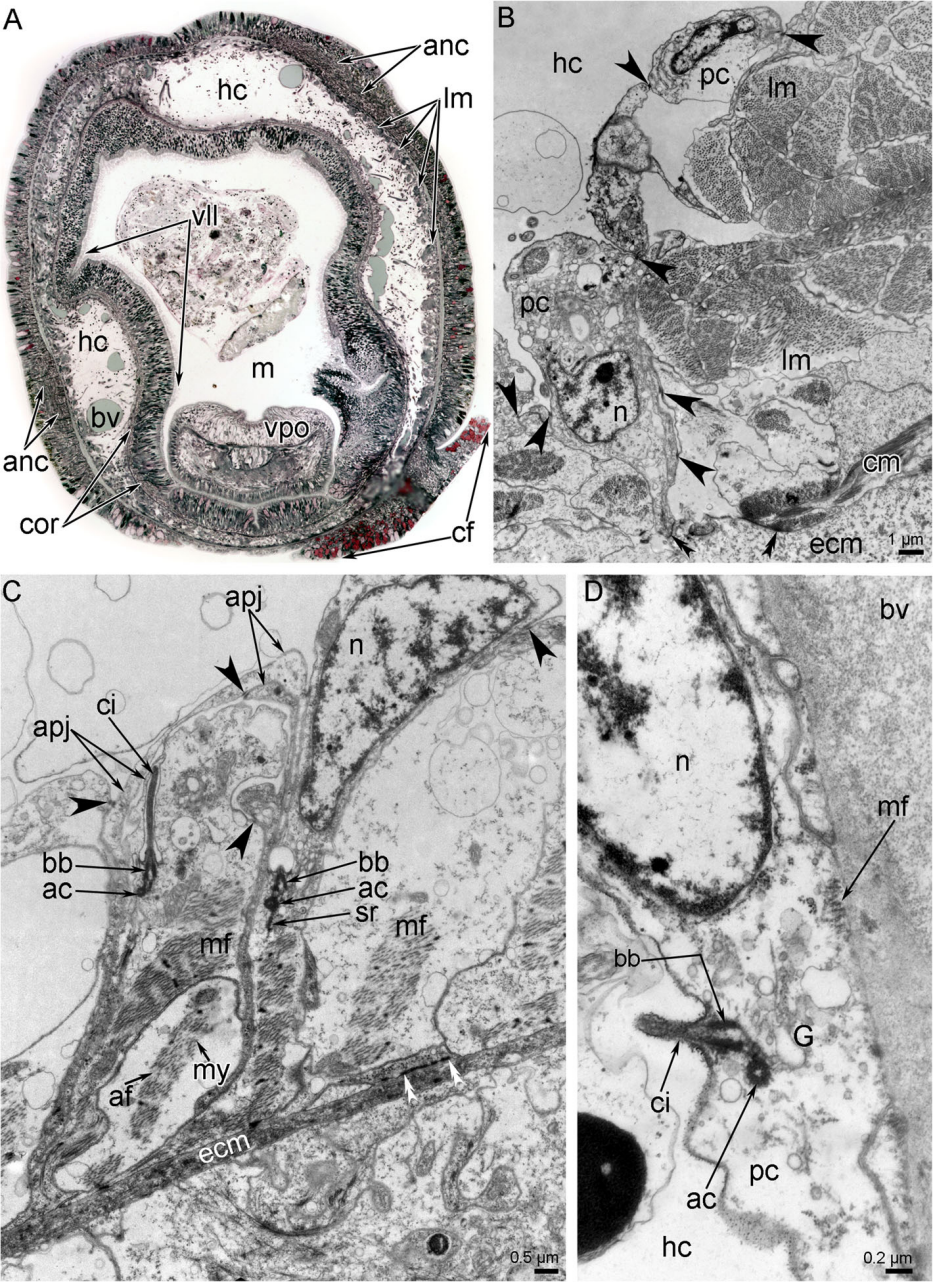
Histology and ultrastructure of the coelom of the head of *Owenia borealis*. **A** Semi-thin transverse section of the head: the spacious head coelom is visible. **B** Ultrastructure of a part of coelomic lining, which is formed by alternating peritoneal and myoepithelial cells. Adherence junctions between cells are indicated by arrowheads. Hemidesmosomes are indicated by double arrowheads. **C** Coelomic lining, which is formed by monociliated myoepithelial cells. Adherence junctions between cells are indicated by arrowheads. Hemidesmosomes are indicated by double arrowheads. **D** A part of wall of blood vessel, which consists of monociliated myoepithelial cells. Abbreviations: ac – accessory centriole; af – actin filaments; anc – outer ring of the anterior nerve center; apj – apical projections of myoepithelial cells; bb – basal body; bv – blood vessel; ci – cilium; cf. – collar fold; cm – circular muscles; cor – circumoral nerve ring of the anterior nerve center; ecm – extracellular matrix; G – Golgi apparatus; hc – head coelom; lm – longitudinal muscle; m – moth; mf – myofilaments; my – myosin filament; n – nucleus; pc – peritoneal cell; sr – striated rootlet; vll – ventrolateral lip; vpo – ventral pharyngeal organ

The coelomic cavity of the tentacles is connected to the voluminous cavity of first body segment (i.e., the head cavity), which is formed by the prostomium and peristomium (Figs. 5A, 6A). The lower border of the head cavity forms ventral and dorsal projections (Fig. 5A). On the ventral side, the head cavity is occupied by a large ventral pharyngeal organ that extends to the lower border of the head cavity (Fig. 5B). On the dorsal side, the head cavity is occupied by voluminous folds of the pharynx (Fig. 5C).

Each tentacle contains a coelomic cavity, which is usually represented by narrow spaces between cells of a coelomic epithelium. The epithelial cells form outgrowths that extend into the cavity and that connect the lining of the coelom of the aboral and oral sides of the tentacles (Figs. 2, 3B). The coelomic lining is composed of myoepithelial cells that form the musculature of the tentacles and the wall of the blood vessel (Figs. 3A, B; 4D). The musculature of each tentacle includes longitudinal, transverse, and oral-aboral muscles (Figs. 2, 3). Transverse muscles form a thin layer that is only present on the oral side of the tentacles (Figs. 2B, 3B). The strands of the longitudinal muscles are much thicker (due to an increased number of cells) on the aboral than on the oral side of the tentacle (Fig. 2B; 3B). Cells of all types of muscles are attached to the extracellular matrix via hemidesmosomes (Fig. 3B). The head cavity is lined by the coelomic epithelium, which is formed by different types of cells (Fig. 6B, C). Most of these cells are myoepithelial monociliated cells that form the longitudinal musculature of the head (Fig. 6A, C). Each myoepithelial monociliated cell bears one long cilium, at the base of which the basal body and accessory centriole are located (Fig. 6C). These cells contact each other via apical adhering junctions, which are usually located on the thin apical projections (Fig. 6C). A large nucleus occupies the apical portion of the cell, whereas myofilaments extend into the basal portion of the cell. Myofilaments extend in basal part of the cells. The cells are anchored to the basal lamina by hemidesmosomes (Fig. 6C). The myoepithelial monociliated cells of the coelomic lining form the walls of blood vessels of the head (Fig. 6D). These cells contain a few basal myofilaments that extend longitudinally (Fig. 6D). The second type of cells of coelomic lining is a typical peritoneal cells, which lack myofilaments and are scattered randomly throughout the epithelium (Fig. 6B). The peritoneal cells are attached to the basal lamina between muscle cells. Peritoneal cells are connected to each other and to the muscle cells by adhering junctions (Fig. 6B).

#### Blood vessels

Three-dimensional modelling revealed that the ventral and dorsal blood vessels give rise to numerous blood vessels in the head and tentacles (Fig. 5D). At the border between the first trunk segment and the head, the ventral blood vessel splits into two lateral efferent branches, which give rise to two prominent lateral blood plexuses (Fig. 5D). The dorsal blood vessel splits into two lateral afferent vessels at the middle of the head (Fig. 5D). In each tentacle, there are three longitudinal blood vessels that are connected to each other (Fig. 5E). Together, they form the tentacular blood plexus.

Myoepithelial cells form the wall of blood vessels (Figs. 3B; 4D). The basal parts of these cells bear myofilaments and form numerous plasmatic projections, which extend into the lumen of the vessel (Fig. 4D).

### Neural elements of the head

#### Anatomy

**Fig. 7:**
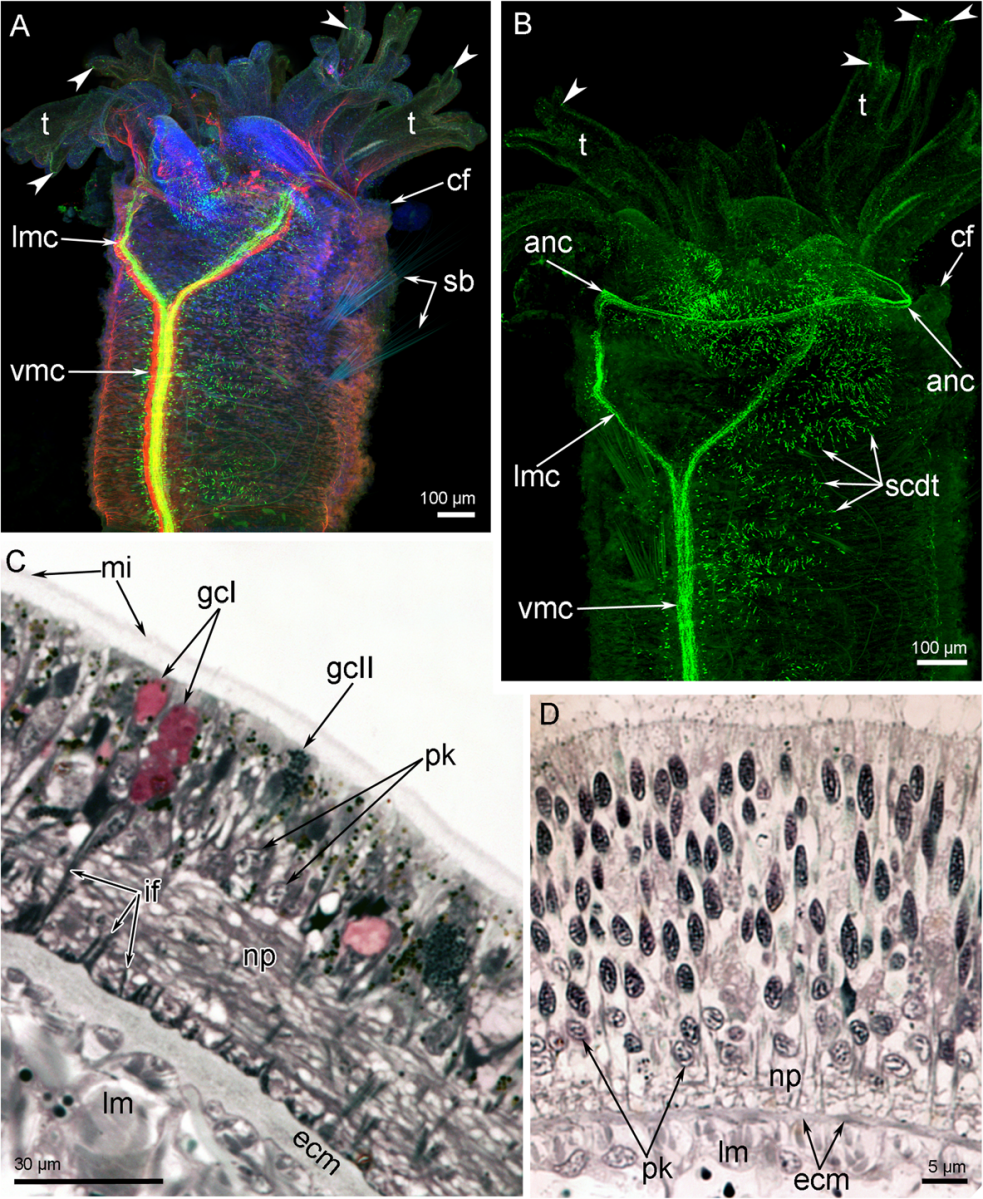
Organization of the nervous system of the head of *Owenia borealis*. **A** General anatomy of the nervous system viewed from the ventral side: Z-projection after double immunostaining against acetylated alpha-tubulin (red) and serotonin (green) and staining with DAPI (blue). **B** Serotonin-lir nerve elements of the head viewed from the ventral side; Z-projection after immunostaining against serotonin (green). Some serotonin-lir cells in the epithelium of tentacles are indicated by arrowheads. **C** Semi-thin transverse section of the outer ring of the anterior nerve center. **D** Semi-thin transverse section of the inner (=circumoral) nerve ring of the anterior nerve center. Abbreviations: anc – outer ring of the anterior nerve center; cf. – collar fold; ecm – extracellular matrix; gcI – gland cell of first type; gcII – gland cell of second type; if – intermediated filaments; lm – longitudinal muscles; lmc – lateral medullary cord; mi – microvilli; np – neuropil; pk – perikarya; sb – setae bundle; scdt – serotonin-lir cells of the digestive tract; t – tentacle; vmc – ventral medullary cord

**Fig. 8:**
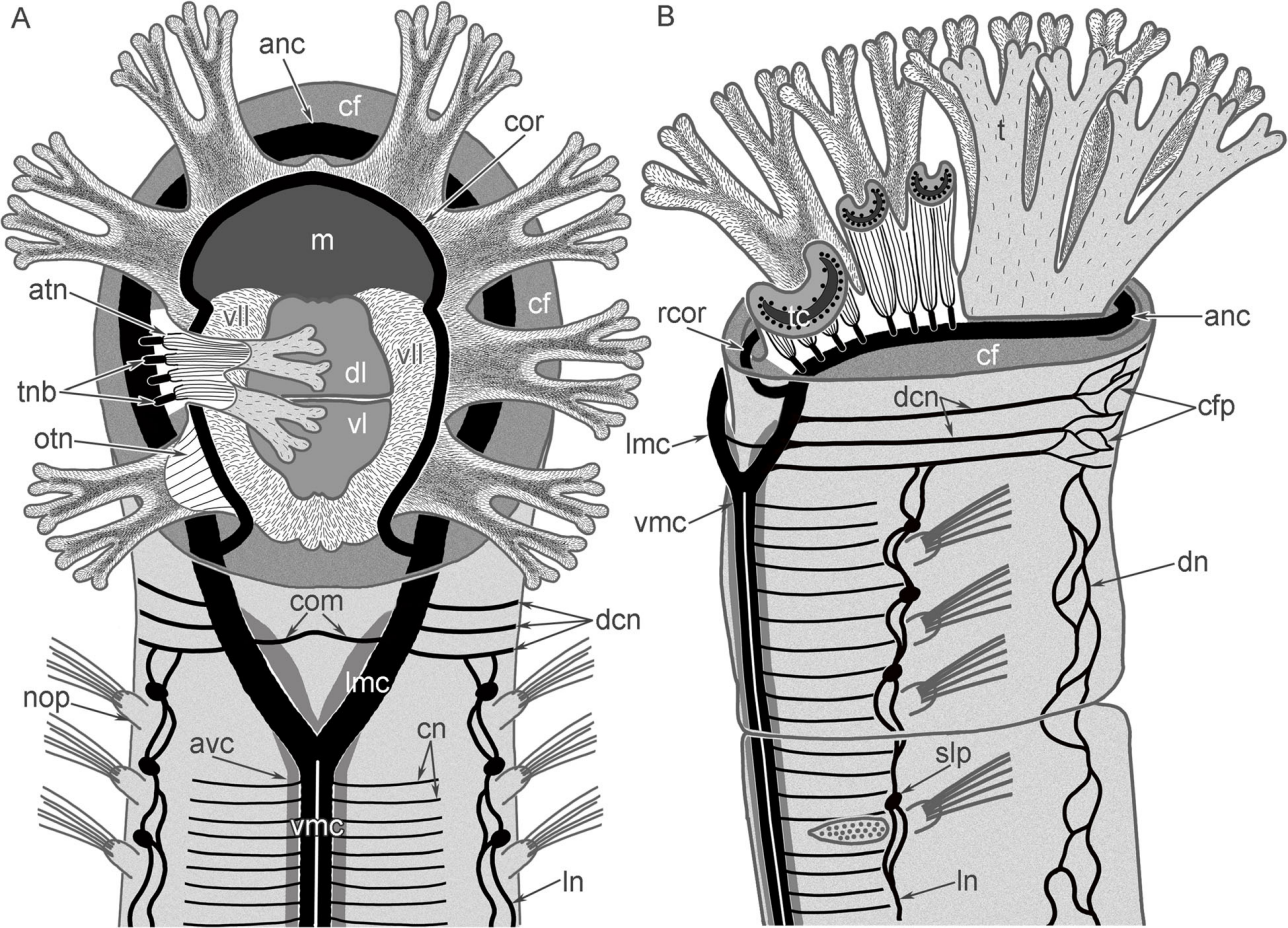
Schemes of the nervous system organization of the anterior body part of *Owenia borealis*. All nerve elements are shown in black. **A** Ventral view. **B** Lateral view. Abbreviations: anc – outer ring of the anterior nerve center; atn – aboral tentacle nerves; avc – peripheral zone of the ventral medullary cord; cf. – collar fold; cfp – collar fold dorsal nerve plexus; cn – cross nerve; com – ventral commissure; cor – inner (=circumoral) nerve ring of the anterior nerve center; dcn – dorsal cross nerve; dl – dorsal lip of the ventral pharyngeal organ; dn – dorsal neurites; ln – lateral nerve; lmc – lateral medullary cord; m – mouth; nep – neuropodia; nop – notopodia; otn – oral tentacle nerves; pbr – posterior portion of the brain; ph – pharynx; rcor – root of circumoral nerve ring; slp – serotonin-like immunoreactive perikarya; t – tentacle; tbn – tentacle neurite bundle; tc – tentacle coelomic cavity; vl – ventral lip of the ventral pharyngeal organ; vmc – ventral medullary cord

The head contains the main elements of the nervous system: the anterior nerve center represented by an outer nerve ring (= medullary dorsal commissure), and an inner (=circumoral) nerve ring, and some nerves, which connect outer and inner nerve rings (Figs. 7A, B; 8A, B). All other nerve elements, such as the ventral medullary nerve cord and the dorsal and lateral neurite bundles, are located in the first and other chaetigers (Fig. 8A, B).

#### Immunocytochemistry

**Fig. 9:**
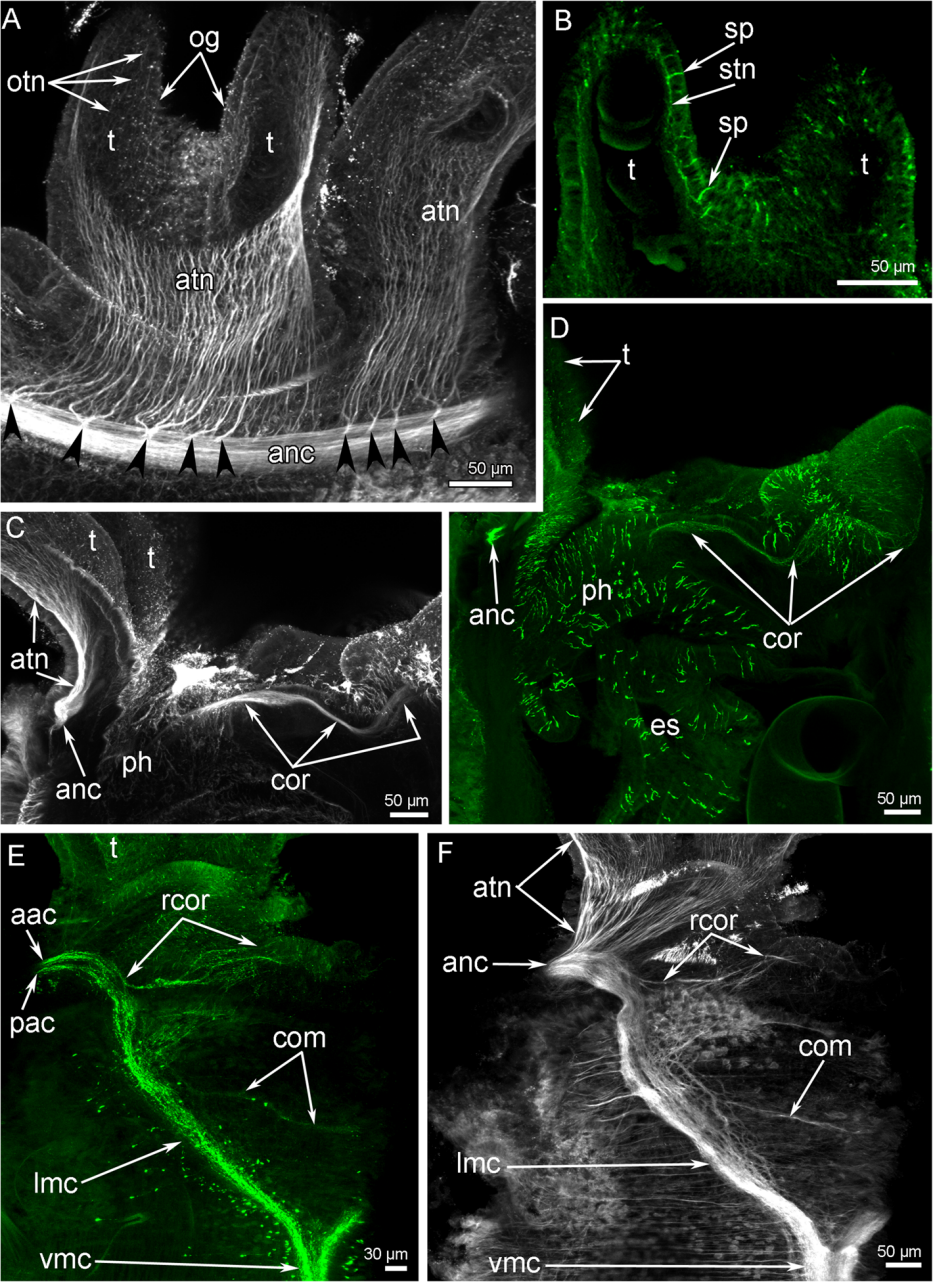
Details of innervation of tentacle crown and oral area in *Owenia borealis*. CLSM data: Z-projections after immunostaining against acetylated alpha-tubulin (grey) and serotonin (green). **A** The aboral side of tentacle base: several short thick nerves (arrowheads) extend from the anterior nerve center and give rise to the oral neurite bundles of tentacles. **B** Serotonin-lir perikarya and neurites in the epithelium of tentacle. **C** Central portion of the head: circumoral nerve ring is visible. **D** Central portion the head: serotonin-lir neurites are visible in the circumoral nerve ring. The epithelium of pharynx contains numerous serotonin-lir cells. **E** A portion of the head viewed from the ventral side. There are anterior and posterior portions of the anterior nerve center. Right ventrolateral root of the circumoral nerve ring and ventral commissure are visible. **F** Right portion of the head viewed from the ventral side. Abbreviations: aac – anterior portion of the outer ring of the anterior nerve center; anc – outer ring of the anterior nerve center; atn – aboral tentacle nerves; com – ventral commissure; cor – inner (=circumoral) ring of the anterior nerve center; es – esophagus; lmc – lateral medullary cord; og – oral groove; otn – oral tentacle nerves; pac – posterior portion of the outer ring of the anterior nerve center; ph – pharynx; rcor – root of circumoral nerve ring; sp. – serotonin-lir perikarya; stn – serotonin-lir neurites of tentacles; t – tentacle; vmc – ventral medullary cord

Many of the neurite bundles exhibit acetylated alpha-tubulin-like immunoreactivity (−lir) (Fig. 9A, C, F) and serotonin-lir (Figs. 7B, 9B, D, E). Interestingly, labelling with both serotonin and acetylated alpha-tubulin antibodies revealed that the outer ring of the anterior nerve center has two parts: an anterior and posterior part (Fig. 9E). The intensity of anti-serotonin antibody staining is similar in the anterior and posterior parts of the nerve center, but an area between the anterior and posterior parts is formed by neurites that do not exhibit serotonin-lir (Fig. 9E).

The outer ring of the anterior nerve center gives rise to the aboral tentacular neurite bundles (Fig. 9A). Four or five thick neurite bundles extend into each arm, where they spilt into many thin neurites that run along the aboral side of the tentacles. Some of these neurites exhibit serotonin-lir and are associated with epidermal sensory cells (Figs. 7A, B; 9B).

**Fig. 10:**
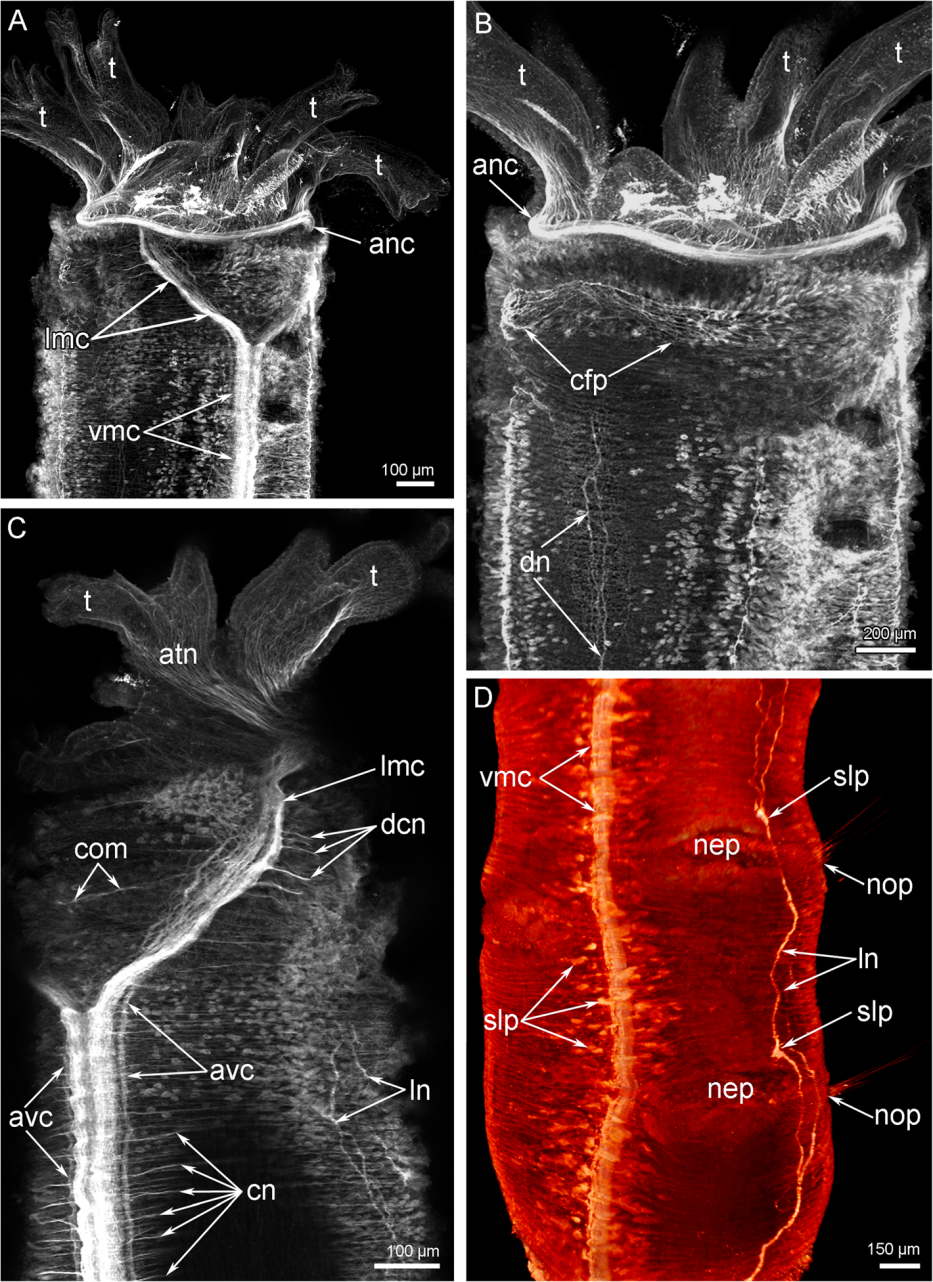
Details of the nerve element location in the head and adjacent segments of *Owenia borealis*. CLSM data: Z-projections (**A**-**C**) after immunostaining against acetylated alpha-tubulin (grey) and volume rendering (**D**) after immunostaining against serotonin (orange). **A** A head with tentacle crown viewed from the ventral side. **B** A head with some tentacles viewed from the dorsal side. **C** Left portion of the head viewed from the ventral side. **D** A portion of the body near the head viewed from the left. Abbreviations: anc – outer ring of the anterior nerve center; atn – aboral tentacle nerves; avc – peripheral zones of the ventral medullary cord; com – ventral commissure; cfp – collar fold dorsal nerve plexus; cn – cross nerve; dcn – dorsal cross nerve; dn – dorsal neurites; lmc – lateral medullary cord; ln – lateral nerve; nep – neuropodia; nop – notopodia; slp – serotonin-like immunoreactive lateral perikarya; t – tentacle; vmc – ventral medullary cord

The outer nerve ring of the anterior nerve center continues into two lateral medullary nerve cords that fuse together on the ventral side at the border between the head and the first chaetiger (Figs. 8A, 10A). Each lateral medullary cord gives rise to a root that skirts a tentacle crown on the oral side and connect the outer and inner (=circumoral) nerve rings of the anterior nerve center (Fig. 9C-F). The circumoral nerve ring gives rise to a few thin neurites that extend along the oral side of the tentacles (Fig. 9A).

On the ventral side of the body, two lateral medullary cords are connected via a single, thin commissure (Figs. 9E, F; 10C). Each lateral medullary cord forms several dorsal neurite bundles that fuse together on the dorsal side to form a dorsal nerve plexus (Fig. 10B, C). Several thin neurites extend along the dorsal side of the body (Fig. 10B). Thin lateral nerve tracts extend from the head region along both lateral sides of the body (Fig. 10D). Above the neuropodium of each segment, these nerve tracts form prominent varicoses (Fig. 10D). The medullary ventral nerve cord consists of two pairs of nerve tracts: left and right (Fig. 10C). In each pair, prominent thick central and thin peripheral zones can be distinguished.

#### Histology and ultastructure

**Fig. 11:**
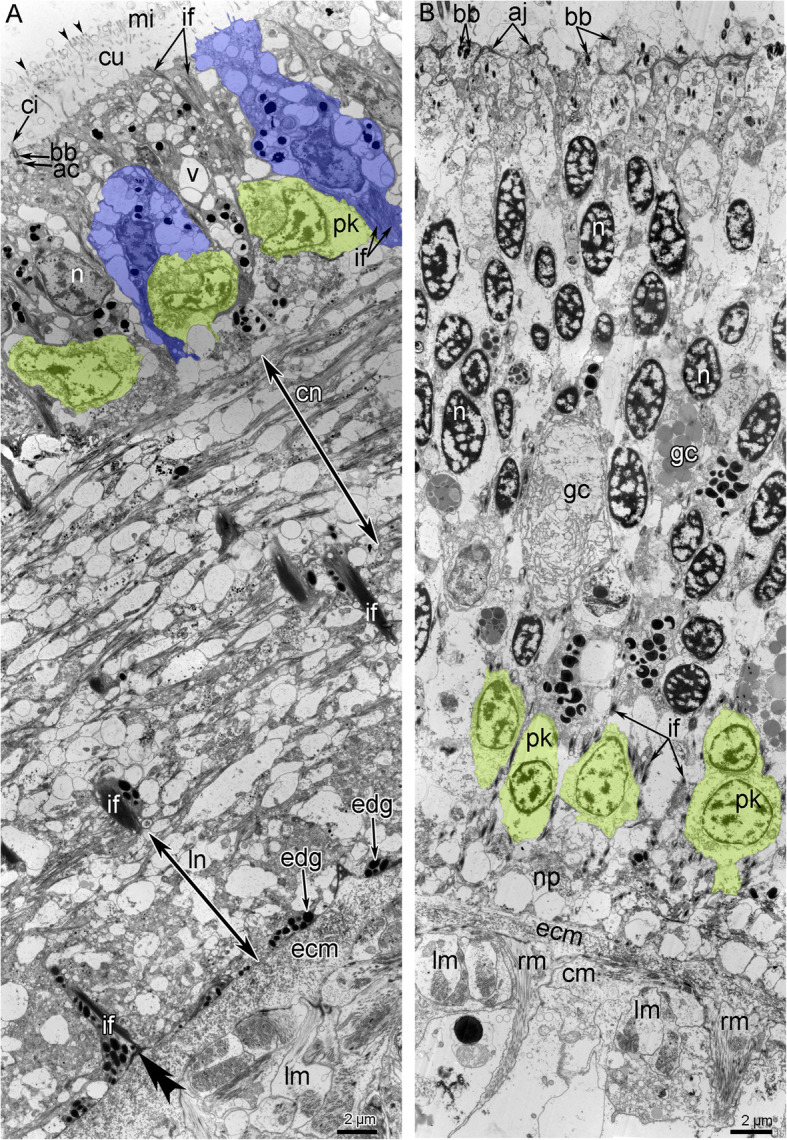
Ultrastructure of head nerve elements in *Owenia borealis*. Ultra-thin transverse sections. **A** A portion of the outer ring of the anterior nerve center. **B** A portion of the circumoral nerve ring. Supportive cells (= radial glial cells) are shown in blue; perikarya are shown in yellow. Hemidesmosome is indicated by double arrowhead. Dense tips of microvilli are indicated by arrowheads. Abbreviations: ac – accessory centriole; aj – adherence junction; bb – basal body; ci – cilium; cm – circular muscles; cn – cross extended neurites; cu – cuticle; ecm – extracellular matrix; edg – electron dense granules; gc – gland cell; if – intermediate filaments; ln – longitudinally extended neurites; mi – microvilli; n – nucleus; np – neuropil; pk – perikaryon; rm. – radial muscles; v –vesicle.

In the head, all nerve elements are located basiepidermally: perikarya and neurite bundles are located between the somata of the epidermal cells and the layer of the extracellular matrix (Figs. 7C, D; 11). As a consequence, each nerve element has a stratified structure, in which the cellular components form three layers, i.e., the upper, middle, and lower layers (Fig. 11). The upper layer is formed by the somata of the epithelial cells. The middle layer is formed by the perikarya of the neurons. The lower layer is formed by nerve projections and projections of epithelial cells (Figs. 7C, D; 11).

The ultrastructure of all elements of outer and inner (=circumoral) nerve rings of the anterior nerve center are similar. The outer ring of the anterior nerve center lies at the tentacle base, in the outer epidermis of the head (Figs. 7C, 11A). The epithelium, which includes the anterior nerve center, is up to 45 μm in height (Figs. 7C, 11A). The neurite bundles, which make up the largest portion of the nerve center, form a layer that is up to 30 μm thick (Fig. 7C). The epithelium, which includes the circumoral nerve ring of the anterior nerve center is up to 43 μm in height; and the neurite bundles form a layer that is about 5 μm thick (Fig. 7D).

According to TEM, the epithelium, which contains the outer ring of the anterior nerve center, is formed by monociliated cells (Fig. 11A), whereas epithelium of circumoral nerve ring is bi- or polyciliated (Fig. 11B). Epithelial cell of both nerve rings have a wide apical part and a narrow basal part that is transformed into a long thin process, in which electron-dense bundles of intermediate filaments extend. The apical surface of the monociliated cells bears thin long microvilli, whose tips are electron dense, and a thick layer of cuticle is located between the microvilli and cilia (Fig. 11A). Epithelial cells of the inner circumoral nerve ring lack thin microvilli and thick layer of cuticle (Fig. 11B).

**Fig. 12:**
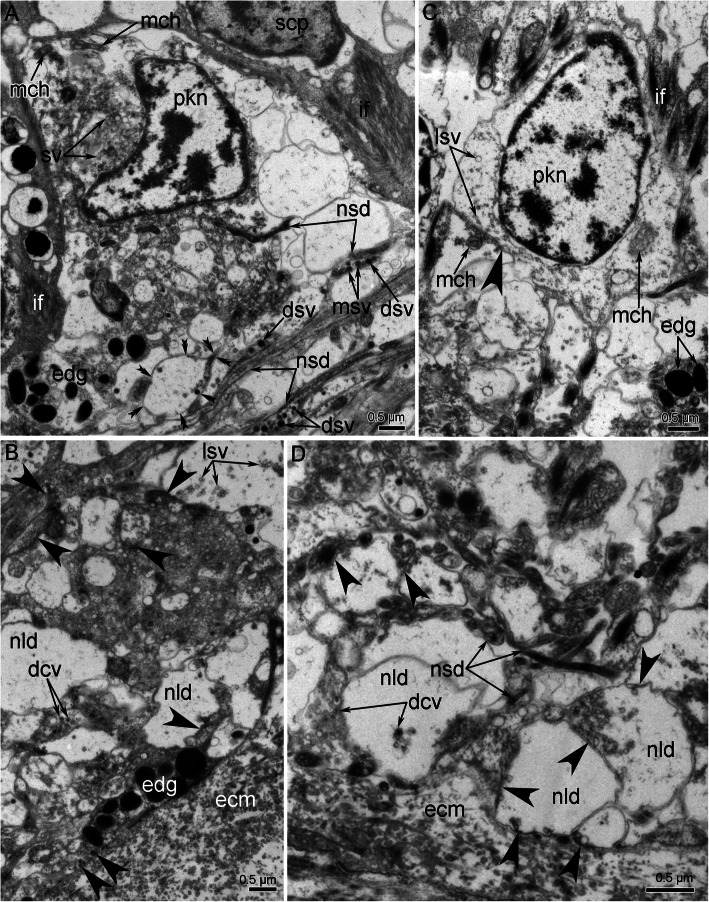
Ultrastructural details of the outer (**A**, **B**) and circumoral nerve rings (**C**, **D**) of the anterior nerve center of *Owenia borealis*. **A**, **C** Perikarya, which are surrounded by supportive cells and their projections. **B**, **D** The basal portions of neuropil. Varicose of neurite of second type is shown by double arrowheads. Synaptic-like structures, which are characterized by cell membrane density and by synaptic vesicle concentration, are indicated by arrowheads. Abbreviations: dcv – dense-core synaptic vesicle; dsv – dense synaptic vesicles; ecm – extracellular matrix; edg – electron dense granules; if – intermediate filaments; lsv – light synaptic vesicle; mch – mitochondrion; msv – synaptic vesicle with content of middle electron density; nld – neurite of large diameter; nsd – neurite of small diameter; pkn – nucleus of perikaryon; scp – supportive cell; sv – synaptic vesicle

The epithelium of the outer ring of the anterior nerve center contains many gland cells of two types, which are similar to the gland cells in the tentacle epithelium: cells of the first type contain large vesicles with mucous content and cells of the send type are filled with small dense granules (Fig. 7C). In both rings of the anterior nerve center, perikarya are scattered between the somata of the epithelial cells, above the neuropil (Fig. 11A, B). These perikarya are small (diameter ~ 5 μm) (Fig. 12A, C). The nucleus in these cells has an irregular shape, lacks a nucleolus, has electron-lucent karyoplasm, and contains large aggregations of heterochromatin in the center (Fig. 12A, C). The cytoplasm of the perikarya contains many synaptic vesicles that differ in diameter and content. The cytoplasm also contains mitochondria that are small, not abundant, and have an electron-dense matrix (Fig. 12A, C). The neuropil, which is formed by numerous neurites, is located between the perikarya and the extracellular matrix (Fig. 12B, D). In the outer ring of the anterior nerve center, two components of the neuropil can be distinguished at the ultrastructural level. The first component is the upper portion of the neuropil, which is mostly formed by circular neurites that are cut longitudinally in transverse sections of the nerve center (Fig. 11A). The second component is the lower layer, which is mostly formed by longitudinal neurites that are cut transversally in transverse sections of the nerve center (Fig. 11A). In the nerve rings, the neuropil consists of neurites that differ in structure. Some of these neurites have small diameters and electron-dense cytoplasm, which regularly form wide varicoses with electron-lucent cytoplasm (Fig. 12A). The cytoplasm of these small neurites contains synaptic vesicles with electron-dense content and with content of intermediate electron density (Fig. 12A, B). Other neurites of the neuropil usually have large diameters and electron-lucent cytoplasm, and contain dense-core synaptic vesicles and vesicles with electron-lucent content (Fig. 12B). In addition to these two types of neurites, the neuropil contains projections of cells that contain ovoid electron-dense granules (Fig. 12B, D).

## Discussion

In the current research, we used TEM and immunocytochemistry coupled with CLSM to study the anatomy and ultra-anatomy of the anterior nerve center of *Owenia borealis.* We also used histology, TEM, SEM, and 3D modelling to examine the organization of the organ system of the tentacle crown with the goal of gaining insight into the evolution of Annelid nervous system and feeding apparatuses.

### The stratified neuroepithelium as a trait of the bilaterian anterior nerve center

The typical brain is regarded as the most anterior ganglion, which comprises a compact central mass of neuropil surrounded by a cell cortex [22]. Previous data [13, 15] and our study revealed the absence of typical ganglionic organization of the anterior nerve center in oweniids: their nerve center does not have a cell cortex and is organized as concentrated intraepidermal nerve plexus.

In general, such organization can be regarded as a stratified neuroepithelium, in which there are three layers: somata of glial cells (epithelial cells), perykaria of neurons, and the neuropil. In glial cells, basal projections are very long and contain intermediate filaments: such a structure is typical for radial glial cells [29]. Interestingly, such a three-layered structure of the stratified neuroepithelium is described in the structure of the intraepidermal brain of magelonids (Fig. 8a in [55]).

Moreover, in many bilaterians, the nerve centers are organized as stratified neuroepithelium. Thus, such organization is described in protostomes (such as brachiopods [56], phoronids [38, 57], oweniid annelids (this study [15];), and in priapulids [29]) and in deuterostomes (such as enteropneust hemichordates [29] and echinoderms [58]), as well as in nemertodermatid acoelomorph belonging to the likely sister group to all remaining bilateria (see Fig. 5F’ in [59]). Such a wide distribution of the stratified neuroepithelium in the nervous system of different bilaterians may indicate that this was the ancestral trait of the anterior nerve center in all bilaterians. That possibility is consistent with earlier suggestions regarding the ancestral state of the intraepidermal and non-ganglionic anterior nerve center of bilaterians [7, 15, 23–29].

### Is the anterior nerve center in oweniids homologous to the dorsal root of the annelid circumesophageal connective?

In the adult oweniids, among two roots of the circumesophageal connectives that are described in the brains of other annelids [23, 60], at least the dorsal root is present. There are two nerve rings in the anterior nerve center in *O. borealis* (here) and in *Galathowenia oculata* [13]. We found the same nerve rings of the anterior nerve center in *O. fusiformis* and in basally branching oweniid taxon *Myriowenia sp.* at histological sections made by Beckers et al. [15] (*O. fusiformis*: sections 15–115: morphdbase.de/?P_Beckers_20170310-M-94.1 and, *Myriowenia sp.*: sections 69–78: morphdbase.de/?P_Beckers_20170310-M-96.1). According to some data [12, 16], in the post-metamorphic stage of *O. fusiformis,* the dorsal root of the circumesophageal connectives contains 5HT-lir and FMRFamide-lir neurites, whereas the ventral root has only 5HT-lir neurites. In the adult oweniids, both the 5HT-lir and FMRFamide-lir nerves are also found in the rings of the anterior nerve center of *G. oculata* [13] and *O. fusiformis* [15]. Thus, based on the immunoreactivity, we suggest the homology of the oweniid anterior nerve center to the dorsal root of the circumesophageal connective of other annelids. Moreover, the outer ring of the anterior nerve center in *O.borealis* has two parts, an anterior and posterior, which could be the ventral and dorsal commissures of the dorsal root of the connectives.

### The non-specialized tentacle crown in oweniids

Oweniids primarily feed on the surfaces of substrates, and those species with a tentacle crown also use it in suspension feeding [61]. The degree of specialization in feeding probably determines the architecture of the tentacle crown, which may differ in number of tentacles, crown length, ramification from the base, rate of branching, and the shape of grooves that conduct particles from the tentacle tips to the mouth [62–66]. As it was shown in our study, the tentacle crown of *O. borealis* also varies in number of arms and tentacles.

#### Ciliation of tentacles

In *O. borealis*, all epithelial cells of tentacles are monociliated. These data are consistent with previous results about *O. fusiformis* [67]. Distal parts of tentacles of *O. borealis* are evenly ciliated and do not bear any ciliated grooves. Oral ciliated groove extend along each arm at its proximal part. This groove is formed due to increase of density of epithelial cells, but not due to appearance of polyciliated cells. In animals with tentacles, usually, the diameter of ciliated grooves defines the size of food particles [63]. *O. borealis* are able to collect particles, which size is up to 20–25 μm, because the ciliated oral groove has the same diameter. The secretion of numerous epithelial gland cells is probably used for agglutination of food particles.

#### Innervation of the tentacle crown

The basally branching oweniid taxon *Myriowenia sp.* has palps as the head appendages that are homologous to palps of the adult Magelonidae [15, 55]. It was suggested earlier [15] that the tentacle crown of *Owenia* species is homologous to palps. On the other hand, the tentacles of *O. borealis* and other *Owenia* species might be comparable with the antennae of other annelids: the antennae (median and lateral) are innervated from the middle and lateral parts of the dorsal roots of the circumesophageal connectives [23, 60]. If we assume that the oweniid anterior nerve center is homologous to the dorsal roots of the circumesophageal connectives of other annelids (see above), then the tentacles of *Owenia* species are likely homologous to the antennae of other annelids.

Accordingly to previous results [15], head appendages of different oweniid species are innervated from the outer nerve ring (ring-shaped brain). Tentacle nerves emanate directly from the anterior nerve center and extend along the aboral side of tentacles [15]. Accordingly to our new data, in *O. borealis*, aboral tentacle neurites originate from thick short neurite bundles, but not directly from the anterior nerve center as do in *O. fusiformis* [15]. Moreover, *O. borealis* has not only outer, but also circumoral nerve ring, which gives rise to the oral tentacle neurite bundles. Thus, tentacles of *O. borealis* are evenly innervated by numerous thin neurite bundles, which are not grouped into prominent nerves. Earlier, the inner nerve ring was shown only in *Galathowenia oculata* [13] and has never been described before in other.

At the same time, two nerve rings are described in different lophophorates, which are highly specialized filter feeders [42, 46, 47, 56]. These nerve rings give rise to the tentacle nerves, which extend along certain zone of each tentacle. In all lophophorates, there are intertentacular nerves, which innervate adjacent tentacles [46]. Among annelids, the highly specialized filter feeders, the sabellids, have intertentacular nerves [61]. These nerves, apparently, allow to coordinated the work of adjacent tentacles and are important for filter feeders, whereas these nerves are not necessary for deposit feeders as oweniids.

#### Musculature of the tentacle crown

The muscles of the tentacle crown in *O. borealis* are mainly represented by longitudinal bundles, most of which are located on the crown’s aboral side. Contraction of the longitudinal muscles pulls the tentacles outward and opens the tentacle crown. The longitudinal musculature in *O. borealis* thereby helps the tentacle crown to collect particles that are suspended in the water column or that have settled on the substrate [61, 63, 68–70]. The reverse folding of the tentacles likely occurs due to the deformed layer of the ECM, which is much thicker on the aboral side than on the oral side of the tentacles. In those annelids that are specialized filter feeders, the outward expansion of the fan of tentacles also occurs due to the contraction of the aboral longitudinal muscles. Those filter feeders, however, also have a cartilaginous skeleton as well as muscles at the base of the tentacular crown that serve as antagonists of the aboral longitudinal muscles, i.e., that enable the organism to withdraw the tentacles and move the captured particles to the mouth [71].

In the tentacles of *O. borealis*, the transverse muscle layer is very thin. Although this muscle layer may represent only short fragments of individual muscle filaments, we suspect that it represents a complete muscle ring. In the tentacles of the specialized filter feeders such as the lophophorates and annelids Sabellidae and Serpulidae, a complete reduction of the transverse muscles occurs [71–74]. That *O. borealis* apparently retains the transverse muscles in the tentacles is consistent with the inference of a non-specialized mode of feeding.

#### Coelomic lining of the tentacle crown

Four types of coelomic myoepithelium have been described in echinoderms, various annelids, and lophophorates: simple, pseudostratified, bipartite pseudostratified, and stratified [75–78]. The coelomic epithelium in *O. borealis* is intermediate between the simple and the pseudostratified myoepithelium. The pseudostratified myoepithelium is known for echinoderms [75, 79], brachiopods [78, 80], phoronids [57, 73], and sedenterian and errantian annelids [76, 77]. The cells of a pseudostratified myoepithelium are arranged in two rows: internal myoepithelial cells and external non-muscular epithelial cells (i.e., cells without myofilaments). In *O. borealis*, both rows of cells have myofilaments. The internal myoepithelial cells are mainly used for contraction, and their wide basal parts contain longitudinal or rarely circular myofilaments (Figs. 3, 6C). Because both types of cells in *O. borealis* are myoepithelial, the pseudostratified myoepithelium of *O. borealis* differs from the pseudostratified myoepithelium described by Rieger and Lombardi [75]. We propose that the coelomic lining of the head and tentacles of *O. borealis* is intermediate between the simple and pseudostratified evolutionary stages of the coelomic epithelium of Bilateria.

The pseudostratified myoepithelium is considered to be associated with the basiepidermal nervous system: each myoepithelial cell receives a signal from neurotransmitters in the immediate vicinity of the neurons [76]. Together, the pseudostratified myoepithelium and the basiepidermal nervous system in *O. borealis* have been recognized as plesiomorphic traits of the epithelia of the body wall in annelids [15, 26, 27] and possibly in Spiralia [7]. It follows that, on the one hand, the myoepithelium of the coelomic lining and the basi-epidermal nervous system co-evolved in *Owenia*. On the other hand, the myoepithelial cells of the coelom do not specialize in performing various functions, all cells carry myofilaments, and there is no typical pseudo-stratified myoepithelium.

To summarize this part of the Discussion, we observed the presence of a non-specialized tentacle crown in *O. borealis* and note that such crowns have also been observed in other oweniids with tentacles. The lack of a specialized tentacle crown corresponds with the structural elements of the nervous, muscular, coelomic, and circulatory systems. *O. borealis* lacks a dorsal brain but instead has an anterior nerve center which might represent the dorsal root of the circumesophageal connectives. There are no intertentacular nerves. Myoepithelial cells of the coelomic cavity are not specialized and represent an intermediate stage between simple and pseudostratified myoepithelium. The blood vessels form a complex network of capillaries in which the afferent and efferent vessels cannot be traced. The transverse muscles remain, and there are no muscles that are antagonistic to the longitudinal muscles that open the tentacle crown.

### Evolution of the tentacle apparatuses

It is assumed that various bilaterians, including annelids, cephalopods, onychophorans, echinoderms, and ascidians, have the same genetic program defining the coordinate grid of the various appendages or outgrowths of the body [81–83]. At the same time, these appendages of the body have different morphologies and perform completely different functions, for example, sensitive perception, nutrition, movement, etc. Interestingly, in different groups of the bilaterians, including annelids, the anterior outgrowths of the body are specialized in capture of the food particles and formed so-called tentacle apparatuses. Here, we consider the evolutionary trends of the organization of tentacular apparatus that are used for suspension and filter feeding.

**Fig. 13:**
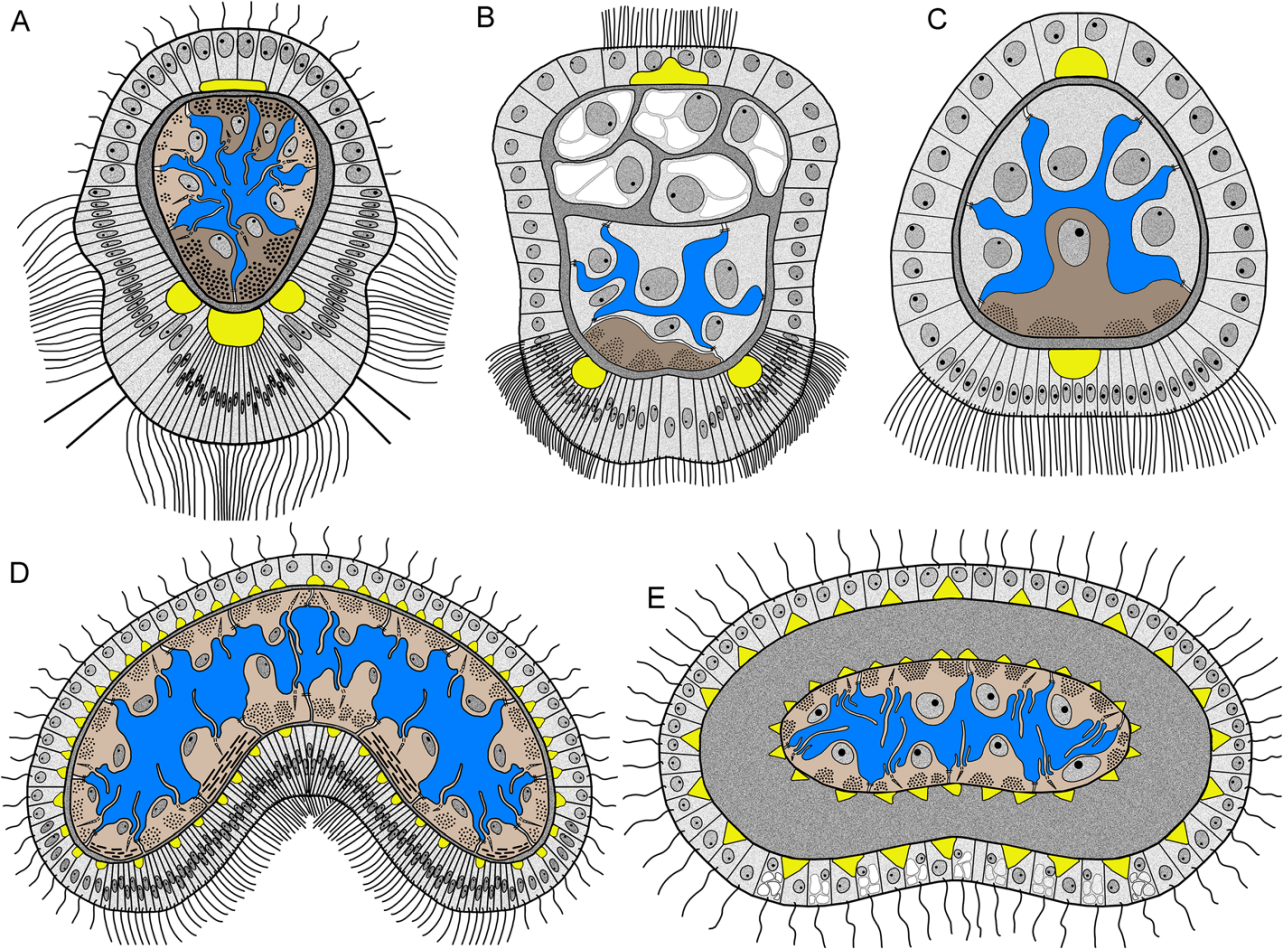
Schemes of transverse section of tentacles of different types. Blood vessel is not shown. Oral side is to the down; aboral side is at the top. **A**, **B** highly specialized tentacles; **C** – less specialized tentacle; **D**, **E** non-specialized tentacles. **A** Generalized tentacle of lophophorates (based on 32,48,51). **B** Generalized tentacle of Serpullidae and Sabellidae (based on 85). **C** Generalized tentacle of Fabriciidae (based on 85) and serpullid *Pomatoceros triqueter* (based on 73). **D** Tentacle of *Owenia borealis* (this study). **E** Tentacle of holothuria *Holothuria forskali* (based on 88). Color code: yellow – nerve elemants; blue – coelomic cavity; light brown – myoepithelial cells; dark brown – mucle cells and myoepithelial cells with numerous myofilaments; dark grey – extracellulart matrix; light grey – epithelial cells

*O. borealis* is one of the various annelids that has a tentacle apparatus or anterior appendages. A comparative analysis of the organization of tentacles in different groups of Bilateria reveals three main patterns of the tentacle specialization (Fig. 13). The first pattern is represented by highly specialized tentacles with a zonality of the epithelium that is co-localized with nerve tracts and muscle bundles. Specialized tentacles are found in some filter feeders including annelids in the families Serpullidae and Sabellidae [84], all lophophorates (Phoronida, Brachiopoda, and Bryozoa) [32, 48, 51], and Kamptozoans (=Entoprocta) [34, 85]. Specialized tentacles always have at least four zones: one oral, one aboral, and two lateral (Fig. 13A, B). Oral and lateral zones are heavily ciliated, whereas cilia are rare or even absent in the aboral zone. The aboral zone can undergo specialization involving the presence of additional skeletal structures and gland cells. These four zones are innervated by different nerve tracts. The epidermal zones and nerve tracts are co-localized. The muscle bundles are also co-localized with certain zones of tentacle. There are usually two muscular bundles, oral and aboral, which allow each tentacle to bend in two directions. Among all filter feeders, lophophorates have the most specialized tentacles, each of which bears eight zones: one frontal (oral), one abfrontal (aboral), two lateral, two laterofrontal, and two lateroabfrontal. Each of these zones is innervated by a specific nerve tract and has a specific function [51].

The second pattern of tentacle organization is less specialized tentacles, which have at least two zones: heavy ciliated oral and less ciliated aboral. In the second pattern, the innervation of each zone is provided by a specific nerve tract. Less specialized tentacles occur in some annelids that are not specialized filter-feeders, i.e., the Fabriciidae and some Serpulidae [72, 84] (Fig. 13C). Although the information is scarce, fabriciids could be deposit and/ or suspension feeders [61, 84, 86].

The third pattern of the tentacle organization is the non-specialized tentacles. Such tentacles lack zonality of the epidermis and co-localization of ciliary cells, nerve tracts, and muscular tracts (Fig. 13D, E). In these non-specialized tentacles, all sides are evenly ciliated. If present, ciliary zones are not co-localized with the nerve tracts or neurite bundles, which are evenly scattered in the tentacle. In the tentacles of the third type, muscle cells do not form bundles, and they are evenly distributed in the tentacle. The non-specialized tentacles can be found in oweniids (this study) and in some holothurians [87]. Holothurians are deposit or suspension feeders and are able to attach deposited particles to tentacles due to the secretion of a glue by gland cells [88–90]. The presence of many gland cells can be regarded as a kind of specialization but cannot be compared with the zonality of the highly specialized tentacles for filter-feeding.

## Conclusions

In this report, we described the anatomy and ultra-anatomy of the tentacular crown of *Owenia borealis*. Because they belong to the clade of palaeoannelids [3, 5], the Oweniidae are important for studies of the morphological traits of annelid ancestors or even of bilaterian metazoan ancestors. In *O. borealis*, the anterior nerve center is represented by stratified neuroepithelium and consists of three layers: apical somata of the glial cells, perikarya of neurons, and the basal neuropil between the thin projections of the glial cells. Based on the available data on the structure of the sister annelid clade Magelonidae and the various bilaterian lineages [15, 29, 38, 55–59], we suggest that the anterior nerve center of the last common ancestor of annelids and possibly of all bilaterian metazoans was composed of a basiepidermal stratified neuroepithelium. Based on the immunoreactivity, we assume that the anterior nerve center of all oweniids is homologous to the dorsal root of the circumesophageal connectives of other annelids [12, 13, 15], as well as the tentacle crown of *Owenia* species are likely homologous to the antennae of other annelids [23, 60]. After describing the architecture of the tentacle crown of *O. borealis*, and its innervation, musculature, blood system, and coelomic myoepithelial lining, we compared the tentacle crown of *O. borealis* with the tentacular apparatuses of the other bilaterian metazoans. These groups have three patterns of tentacle organization: highly specialized tentacles, less specialized tentacles, and non-specialized tentacles. Our anatomical and ultra-anatomical data suggest that *O. borealis* has the least specialized tentacle apparatus, whose type of organization may reflect the ancestral pattern of tentacles in bilaterians.

### Data availability statement

The data sets analyzed during this study are available from ET upon request.

## References

[1] Schmidt-Rhaesa A. The evolution of organ systems: Oxford University Press; 2007.

[2] Struck TH, Paul C, Hill N, Hartmann S, Hosel C, Kube M (2011). Phylogenomic analyses unravel annelid evolution. Nature..

[3] Struck TH, Golombek A, Weigert A, Franke FA, Westheide W, Purschke G, et al. The Evolution of Annelids Reveals Two Adaptive Routes to the Interstitial Realm. Curr Biol. 2015:1–7. 10.1016/j.cub.2015.06.007 26212885

[4] Weigert A, Helm C, Meyer M, Nickel B, Arendt D, Hausdorf B (2014). Illuminating the base of the annelid tree using transcriptomics. Mol Biol Evol.

[5] Weigert A, Bleidorn C (2016). Current status of annelid phylogeny. Org Divers Evol.

[6] Andrade SCS, Novo M, Kawauchi GY, Worsaae K, Pleijel F, Giribet G (2015). Articulating “ archiannelids ”: Phylogenomics and annelid relationships , with emphasis on meiofaunal taxa. Mol Biol Evol.

[7] Helm C, Beckers P, Bartolomaeus T, Drukewitz SH, Kourtesis I, Weigert A (2018). Convergent evolution of the ladder-like ventral nerve cord in Annelida. Front Zool.

[8] Wilson D (1932). On the mitraria larva of *Owenia fusiformis* Delle Chiaje. Philos Trans R Soc London, Ser B.

[9] Liwanow N, Porfirjewa N (1967). Die Organisation der Pogonophoren und deren Beziehungen zu den Polychäten. Biol Zent Bl.

[10] Lagutenko Y (1987). Synaptic terminations on basal lamina of epidermis of oweniids (Polychaeta, Oweniidae). Proc Acad Sci USSR.

[11] Lagutenko Y (1993). The ultrastructure of synapses in the primitive intraepidermal nervous system of *Myriochele oculata* Zachs (Polychaeta, Oweniidae). Tsitologiya..

[12] Helm C, Vöcking O, Kourtesis I, Hausen H (2016). Owenia fusiformis–a basally branching annelid suitable for studying ancestral features of annelid neural development. BMC Evol Biol.

[13] Rimskaya-Korsakova NN, Kristof A, Malakhov VV, Wanninger A (2016). Neural architecture of *Galathowenia oculata* Zach, 1923 (Oweniidae, Annelida). Front Zool.

[14] Rimskaya-Korsakova N, Dyachuk V, Temereva E (2020). Parapodial glandular organs in *Owenia borealis* (Annelida: Oweniidae) and their possible relationship with nephridia. J Exp Zool Part B Mol Dev Evol.

[15] Beckers P, Helm C, Purschke G, Worsaae K, Hutchings P, Bartolomaeus T (2019). The central nervous system of Oweniidae (Annelida) and its implications for the structure of the ancestral annelid brain. Front Zool.

[16] Carrillo-Baltodano AM, Seudre O, Guynes K, Martín-Durán JM (2021). Early embryogenesis and organogenesis in the annelid *Owenia fusiformis*. Evodevo.

[17] Erwin DH. The developmental origins of animal bodyplans. In: Neoproterozoic geobiology and paleobiology: Springer; 2006. p. 159–97.

[18] Northcutt RG (2012). Evolution of centralized nervous systems: two schools of evolutionary thought. Proc Natl Acad Sci.

[19] Hirth F, Kammermeier L, Frei E, Walldorf U, Noll M, Reichert H (2003). An urbilaterian origin of the tripartite brain: developmental genetic insights from *Drosophila*. Development..

[20] Arendt D, Tosches MA, Marlow H (2016). From nerve net to nerve ring, nerve cord and brain—evolution of the nervous system. Nat Rev Neurosci.

[21] Annelida BT, Bullock T, Horridge G (1965). Structure and function in the nervous system of invertebrates.

[22] Richter S, Loesel R, Purschke G, Schmidt-rhaesa A, Scholtz G, Stach T (2010). Invertebrate neurophylogeny : suggested terms and definitions for a neuroanatomical glossary. Front Zool.

[23] Purschke G, Schmidt-Rhaesa A, Harzsch S, Purschke G (2015). Annelida: basal groups and Pleistoannelida. Structure and evolution of invertebrate nervous systems.

[24] Drasche R von. 2. Anatomie von Owenia filiformis. - Wien. 22 pp. In: Beiträge zur feineren Anatomie der Polychaeten Zweites Heft: Anatomie von Owenia fusiformis delle Chiaje. C. Gerold’. Wien; 1885. p. 1–22.

[25] McIntosh W (1917). On the nervous system and other points in the structure of *Owenia* and *Myriochele*. Ann Mag Nat Hist, Ser.

[26] Bubko O, Minichev Y (1972). Nervous system of Oweniidae (Polychaeta). Zool zhurnal.

[27] Lagutenko YP (2002). Early forms of evolution of the basiepidermal nerve plexus of bilateria as a possible evidence for primary diversity of its initial state. J Evol Biochem Physiol.

[28] Hejnol A, Lowe CJ (2015). Embracing the comparative approach: how robust phylogenies and broader developmental sampling impacts the understanding of nervous system evolution. Philos Trans R Soc B Biol Sci.

[29] Helm C, Karl A, Beckers P, Kaul-Strehlow S, Ulbricht E, Kourtesis I (1859). Early evolution of radial glial cells in Bilateria. Proc R Soc B Biol Sci.

[30] Martín-Durán JM, Hejnol A (2021). A developmental perspective on the evolution of the nervous system. Dev Biol.

[31] Brusca RC, Moore W, Shuster SM (2016). Invertebrates (3 rd еdn).

[32] Strathmann R (1973). Function of lateral cilia in suspension feeding of lophophorates (Brachiopoda, Phoronida, Ectoprocta). Mar Biol.

[33] Nielsen C (1987). Structure and function of metazoan ciliary bands and their phylogenetic significance. Acta Zool.

[34] Riisgård HU, Nielsen C, Larsen PS (2000). Downstream collecting in ciliary suspension feeders: the catch-up principle. Mar Ecol Prog Ser.

[35] Riisgård HU, Larsen PS (2001). Minireview: Ciliary filter feeding and bio-fluid mechanics—present understanding and unsolved problems. Limnol Oceanogr.

[36] Adrianov AV, Malakhov VV, Maiorova AS (2006). Development of the tentacular apparatus in sipunculans (Sipuncula): I. *Thysanocardia nigra* (Ikeda, 1904) and *Themiste pyroides* (Chamberlin, 1920). J Morphol.

[37] Kuzmina TV, Malakhov VV (2007). Structure of the brachiopod lophophore. Paleontol J.

[38] Temereva EN, Malakhov VV (2009). On the organization of the lophophore in phoronids (Lophophorata: Phoronida). Russ J Mar Biol.

[39] Temereva EN, Tsitrin EB (2015). Modern data on the innervation of the lophophore in *Lingula anatina* (Brachiopoda) support the monophyly of the lophophorates. PLoS One.

[40] Temereva EN, Kosevich IA (2016). The nervous system of the lophophore in the ctenostome *Amathia gracilis* provides insight into the morphology of ancestral ectoprocts and the monophyly of the lophophorates. BMC Evol Biol.

[41] Temereva EN, Kuzmina TV (2017). The first data on the innervation of the lophophore in the rhynchonelliform brachiopod Hemithiris psittacea: what is the ground pattern of the lophophore in lophophorates?. BMC Evol Biol.

[42] Temereva EN (2017). Innervation of the lophophore suggests that the phoronid *Phoronis ovalis* is a link between phoronids and bryozoans. Sci Rep.

[43] Temereva EN (2019). Myoanatomy of the lophophore in adult phoronids and the evolution of the phoronid lophophore. Biol Bull.

[44] Temereva EN (2019). Myoanatomy of the phoronid *Phoronis ovalis*: functional and phylogenetic implications. Zoology..

[45] Kuzmina TV, Temereva EN (2019). Organization of the lophophore in the deep-sea brachiopod *Pelagodiscus atlanticus* and evolution of the lophophore in the Brachiozoa. Org Divers Evol.

[46] Temereva EN (2020). Novel data on the innervation of the lophophore in adult phoronids (Lophophorata, Phoronida). Zoology..

[47] Isaeva MA, Kosevich IA, Temereva EN. Peculiarities of Tentacle Innervation of *Flustrellidra hispida* and Evolution of Lophophore in Bryozoa. In: Doklady Biological Sciences: Springer; 2021. p. 30–3. 10.1134/S0012496621010038 33635487

[48] Santagata S. Phoronida. In: Evolutionary developmental biology of invertebrates 2: Springer; 2015. p. 231–45.

[49] Santagata S. Brachiopoda. In: Evolutionary developmental biology of invertebrates 2: Springer; 2015. p. 263–77.

[50] Santagata S. Ectoprocta. In: Evolutionary Developmental Biology of Invertebrates 2: Springer; 2015. p. 247–62.

[51] Temereva EN (2020). First data on the organization of the nervous system in juveniles of *Novocrania anomala* (Brachiopoda, Craniiformea). Sci Rep.

[52] Capa M, Parapar J, Hutchings P (2012). Phylogeny of Oweniidae (Polychaeta) based on morphological data and taxonomic revision of Australian fauna. Zool J Linnean Soc.

[53] Koh B-S, Bhaud MR, Jirkov IA (2003). Two new species of *Owenia* (Annelida: Polychaeta) in the northern part of the North Atlantic Ocean and remarks on previously erected species from the same area. Sarsia North Atl Mar Sci.

[54] Schneider CA, Rasband WS, Eliceiri KW (2012). NIH Image to ImageJ: 25 years of image analysis. Nat Methods.

[55] Beckers P, Helm C, Bartolomaeus T (2019). The anatomy and development of the nervous system in Magelonidae (Annelida) - Insights into the evolution of the annelid brain. BMC Evol Biol.

[56] Kuzmina T, Temereva E (2021). Ultrastructure of ganglia in the brachiopod *Coptothyris grayi* and its phylogenetic significance. J Zool Syst Evol Res.

[57] Temereva EN, Schmidt-Rhaesa A, Harzsch S, Purschke G (2015). Phoronida. Structure and evolution of invertebrate nervous systems.

[58] Mashanov V, Zueva O, Rubilar T, Epherra L, García-Arrarás JE, Schmidt-Rhaesa A, Harzsch S, Purschke G (2015). Echinodermata. Structure and evolution of invertebrate nervous systems.

[59] Børve A, Hejnol A (2014). Development and juvenile anatomy of the nemertodermatid *Meara stichopi* (Bock) Westblad 1949 (Acoelomorpha). Front Zool.

[60] Orrhage L, Müller MCM (2005). Morphology of the nervous system of Polychaeta (Annelida). Hydrobiologia..

[61] Jumars PA, Dorgan KM, Lindsay SM (2015). Diet of worms emended: an update of polychaete feeding guilds.

[62] Hansen A (1882). Recherches sur les annélides recueillies par M. le professeur Édouard van Benedon pendant son voyage au Brésil et à la Plata. Mémoires Couronnes Mémoires des Savants Etrang publiés par L’Académie R des Sci des Lettres des B-art Belgique.

[63] Phillips DR (1957). The feeding mechanism and structure of the gut of *Owenia fusiformis* delle Chiaje. J Mar Biol Assoc United Kingdom.

[64] Ford E, Hutchings P (2005). An analysis of morphological characters of *Owenia* useful to distinguish species: description of three new species of *Owenia* (Oweniidae: Polychaeta) from Australian waters. Mar Ecol.

[65] Silva L, Lana P (2018). Strategies for tube construction in *Owenia caissara* (Oweniidae, Annelida) from southern Brazil. Zoology..

[66] Díaz-Díaz O, Parapar J, Moreira J (2018). A new species of genus *Owenia* Delle-Chiaje, 1844 (Annelida; Oweniidae) from the coast of Venezuela. Cah Biol Mar.

[67] Golding DW, Gardiner SL, Harrison FW (1992). Polychaeta: nervous system. Microscopic anatomy of invertebrates.

[68] Eckman JE, Nowell ARM, Jumars PA (1981). Sediment destabilization by animal tubes. J Mar Res.

[69] Nowell ARM, Jumars PA, Self RFL, Southard JB. The effects of sediment transport and deposition on infauna: results obtained in a specially designed flume. In: Ecology of marine deposit feeders: Springer; 1989. p. 247–68.

[70] Self RFL, Jumars PA (1988). Cross-phyletic patterns of particle selection by deposit feeders. J Mar Res.

[71] Nicol E (1931). The Feeding Mechanism, Formation of the Tube, and Physiology of Digestion in *Sabella pavonina*. Earth Environ Sci Trans R Soc Edinburgh.

[72] Hanson J (1949). Observations on the branchial crown of the Serpulidae (Annelida, Polychaeta). J Cell Sci.

[73] Pardos F, Roldan C, Benito J, Emig CC (1991). Fine structure of the tentacles of *Phoronis australis* Haswell (Phoronida, Lophophorata). Acta Zool.

[74] Temereva EN, Tsitrin EB (2013). Development, organization, and remodeling of phoronid muscles from embryo to metamorphosis (Lophotrochozoa: Phoronida). BMC Dev Biol.

[75] Rieger RM, Lombardi J (1987). Ultrastructure of coelomic lining in echinoderm podia: significance for concepts in the evolution of muscle and peritoneal cells. Zoomorphology..

[76] Fransen ME, Ax P (1988). Coelomic and vascular systems. The ultrastructure of Polychaeta Microfauna marina.

[77] Bartolomaeus T, Ax P (1994). On the ultrastructure of the coelomic lining in the Annelida, Sipuncula and Echiura. Microfauna Marina.

[78] Temereva EN (2017). Ultrastructure of the coelom in the brachiopod *Lingula anatina*. J Morphol.

[79] Gardiner SL, Rieger RM (1980). Rudimentary cilia in muscle cells of annelids and echinoderms. Cell Tissue Res.

[80] Kuzmina TV, Temereva EN, Malakhov VV (2018). Ultrastructure of the lophophoral coelomic lining in the brachiopod *Hemithiris psittacea*: functional and evolutionary significance. Zoomorphology..

[81] Panganiban G, Irvine SM, Lowe C, Roehl H, Corley LS, Sherbon B (1997). The origin and evolution of animal appendages. Proc Natl Acad Sci U S A.

[82] Tarazona OA, Lopez DH, Slota LA, Cohn MJ (2019). Evolution of limb development in cephalopod mollusks. Elife.

[83] Shubin N, Tabin C, Carroll S (1997). Fossils, genes and the evolution of animal limbs. Nature..

[84] Tilic E, Rouse GW, Bartolomaeus T (2021). Comparative ultrastructure of the radiolar crown in Sabellida (Annelida). Zoomorphology..

[85] Borisanova AO (2020). Two types of the tentacle structure of Entoprocta and the fine structure of the vestibular groove. Zoomorphology.

[86] Zhadan A, Vortsepneva E, Tzetlin A (2015). Ontogenetic development and functioning of the anterior end of *Cossura pygodactylata* Jones, 1956 (Annelida: Cossuridae). Zoomorphology..

[87] Bouland C, Massin C, Jangoux M (1982). The fine structure of the buccal tentacles of *Holothuria forskali* (Echinodermata, Holothuroidea). Zoomorphology..

[88] Fankboner PV, Cameron JL (1985). Seasonal atrophy of the visceral organs in a sea cucumber. Can J Zool.

[89] Foster GG, Hodgson AN (1996). Feeding, tentacle and gut morphology in five species of southern African intertidal holothuroids (Echinodermata). African Zool.

[90] Graham ER, Thompson JT (2009). Deposit-and suspension-feeding sea cucumbers (Echinodermata) ingest plastic fragments. J Exp Mar Bio Ecol.

[91] Gardiner SL (1978). Fine Structure of the Ciliated Epidermis on the Tentacles of *Owenia fusiformis* (Polychaeta, weniidae). Zoomorphologie..

